# Review of sustainable, eco-friendly, and conductive polymer nanocomposites for electronic and thermal applications: current status and future prospects

**DOI:** 10.1186/s11671-024-03965-2

**Published:** 2024-02-19

**Authors:** Elnaz Tamjid, Parvin Najafi, Mohammad Amin Khalili, Negar Shokouhnejad, Mahsa Karimi, Nafise Sepahdoost

**Affiliations:** 1 Department of Nanobiotechnology, Faculty of Biological Sciences, Tarbiat Modares University, P.O. Box 14115-154, Tehran, Iran; 2 Department of Biomaterials, Faculty of Interdisciplinary Science and Technology, Tarbiat Modares University, P.O. Box 14115-154, Tehran, Iran; 3 Faculty of Engineering and Natural Sciences, Tampere University, 33720 Tampere, Finland; 4 Department of Biomaterials, Faculty of Biological Sciences, Tarbiat Modares University, P.O. Box 14115-154, Tehran, Iran

**Keywords:** Functional materials, Nanofillers, Nanocomposites, Thermal stability and conductivity, Electrical conductivity

## Abstract

Biodegradable polymer nanocomposites (BPNCs) are advanced materials that have gained significant attention over the past 20 years due to their advantages over conventional polymers. BPNCs are eco-friendly, cost-effective, contamination-resistant, and tailorable for specific applications. Nevertheless, their usage is limited due to their unsatisfactory physical and mechanical properties. To improve these properties, nanofillers are incorporated into natural polymer matrices, to enhance mechanical durability, biodegradability, electrical conductivity, dielectric, and thermal properties. Despite the significant advances in the development of BPNCs over the last decades, our understanding of their dielectric, thermal, and electrical conductivity is still far from complete. This review paper aims to provide comprehensive insights into the fundamental principles behind these properties, the main synthesis, and characterization methods, and their functionality and performance. Moreover, the role of nanofillers in strength, permeability, thermal stability, biodegradability, heat transport, and electrical conductivity is discussed. Additionally, the paper explores the applications, challenges, and opportunities of BPNCs for electronic devices, thermal management, and food packaging. Finally, this paper highlights the benefits of BPNCs as biodegradable and biodecomposable functional materials to replace traditional plastics. Finally, the contemporary industrial advances based on an overview of the main stakeholders and recently commercialized products are addressed.

## Introduction

Biodegradable polymer nanocomposites (BPNCs) are two-phase materials that contain evenly dispersed organic or inorganic fillers as additions of at least one dimension within the nanometer range, specifically ranging from 1 to 100 nm. Micro composites are surpassed by BPNCs in terms of both surface area and aspect ratio, which improves several essential features. The biodegradable polymeric matrices include various compositions, either derived from petroleum or biological sources. Recycling common polymers such as polyethylene (PE), polypropylene (PP), polystyrene (PS), polyvinylidene fluoride (PVDF), polyurethane, and polymethylmethacrylate (PMMA) is difficult since they are non-biodegradable. Economic and environmental concerns encourage the application of biodegradable polymers derived from sustainable resources such as biomass to reduce the risks of plastic residues in the environment. Thus, the potential of BPNCs in the industrial sector relies on better recyclability, rapid processability, enhanced physical, mechanical, and chemical properties, and low cost [1–3]. Their replacement, on the other hand, faces notable challenges, such as brittleness, limited thermal stability, and poor barrier characteristics. The incorporation of nanofillers into polymeric matrices not only improves the performance of BPNCs but also has an important effect on the elevation of their biodegradability. Nonetheless, It is imperative to acknowledge that no individual biodegradable polymer possesses the capability to fulfill all necessary criteria for a specific application [3–5].

In the past few years, there has been a tendency to fabricate multi-component polymer composites for the purpose of producing distinct multifunctional materials. Specific BPNCs can be made with various polymer matrices, fillers, and manufacturing methods [6]. Advancements in biodegradable polymers have led to innovative manufacturing techniques that make it possible to commercialize BPNCs, with the entire process optimized from raw materials to finished products [7–9].

The dielectric, thermal, and electrical conductivity properties of BPNCs are essential for their use in the electronics sectors. The frequent upgrade of portable electronic devices such as smartphones and tablets has increased the production of electronic waste. Conductive polymer composites have various superior properties, such as high electrical conductivity, specific strength, specific modulus, flexibility, and thermal properties, besides biodegradability. Therefore, recycling conductive materials can meet specific requirements [10–14]. For example, carbon fiber composites have high modulus and corrosion resistance, but their disposal poses a risk of environmental pollution. Hence, using BPNCs as conductive polymer composites in biodegradable electronics can be a promising solution to this problem due to their environmental safety and disposability. This has become an international research trend [15–17]. One of the major challenges for such a transformation is the lack of thermal and/or electrical conductive biodegradable plastics. A promising solution is to fabricate BPNCs with good thermal and/or electrical conductivity. A practical way to enhance the conductivity of BPNCs is to create a network structure based on highly conductive additives/fillers within the polymer matrix. However, the thermal and electrical conductivity are contingent upon the intrinsic conductivity of the fillers and their interactions with the polymer matrix, which can limit the filler concentration and thus the achievable thermal or electrical conductivity [18, 19]. Accordingly, in recent times, scholarly investigations have been directed towards the examination of adding nanofillers with excellent properties to polymers to enhance their performance as substrates, insulators, semiconductors, conductors, dielectrics, and electronic packaging.

This review article presents a comprehensive overview of the developments of BPNCs, their dielectric, thermal, and electrical conductivity properties, the main characterization methods, and their potential applications. It also highlights the latest progress in technology transfer by some renowned companies.

## Dielectric studies

### Dielectric properties

The dielectric properties are essential for applications such as microelectronics, optoelectronics, biomedical devices, and packaging materials [20]. BPNCs are a new class of hybrid materials with wide technological applications due to their improved and adjustable dielectric, thermal, and electrical properties. These properties depend on the polymer matrix, nanofiller types, and their concentrations. BPNCs also have low cost, excellent flexibility, lightweight, and easy fabrication methods. Because of environmental pollution and the high costs of managing non-biodegradable materials, biodegradable polymers derived from renewable or natural resources are progressively supplanting synthetic or petroleum-based polymers [21–25]. The dielectric properties of a polymer matrix are influenced by the atomic, electronic, interfacial, and orientation polarization of the constituent molecules [26]. The nanofiller distribution and interface adhesion strength greatly affect the thermal, physical, dielectric, and electrical properties of a BPNC. To improve and optimize these properties, the nanofiller and polymer matrix should have good compatibility and interaction [27].

### Dielectric properties measurement

**Fig. 1:**
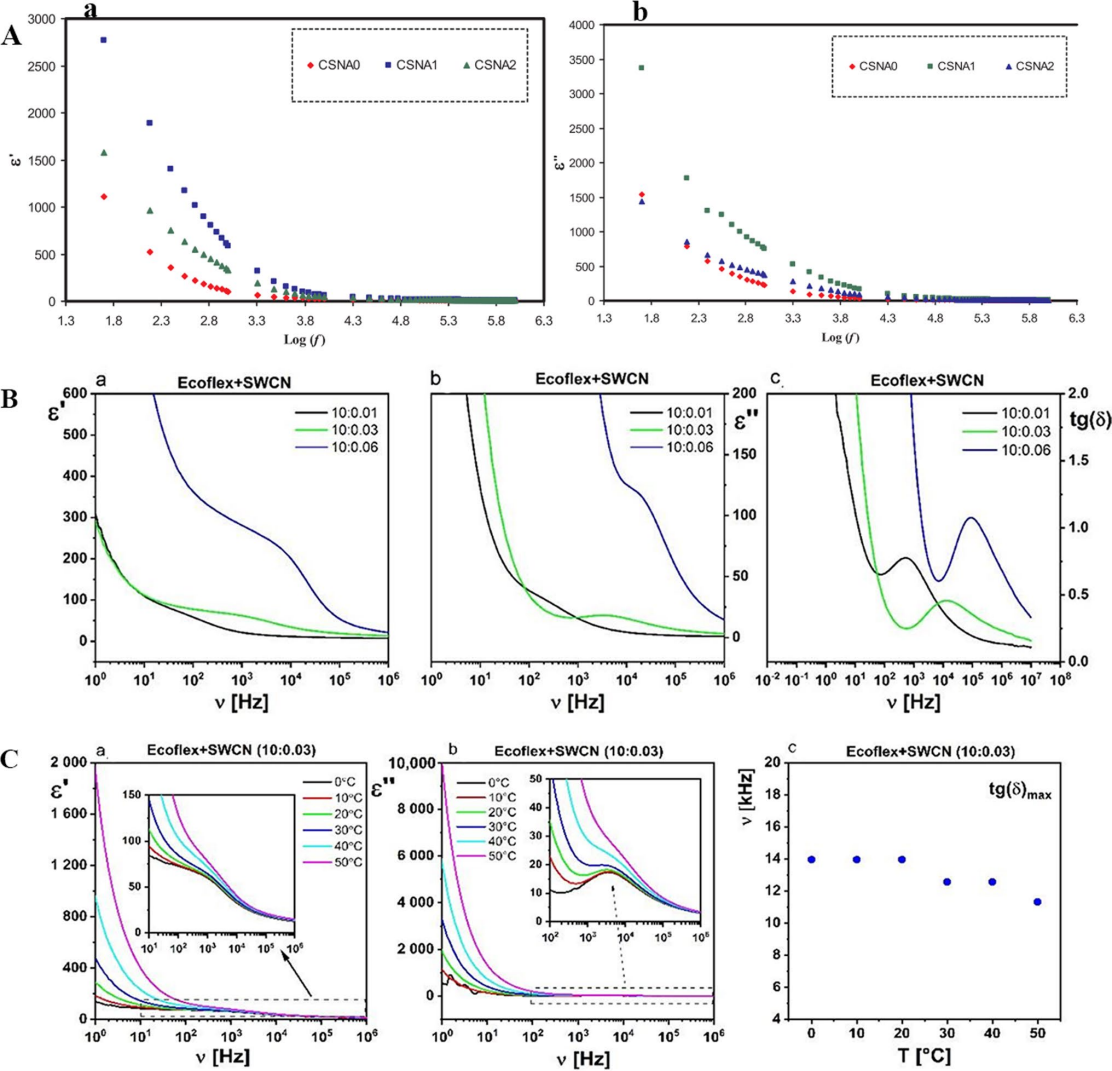
**A** The relationship between (*a*) the dielectric constant and (*b*) dielectric loss at different frequencies is shown for the samples labeled CSNA0, CSNA1, and CSNA2. These samples contain 0%, 1%, and 3% weight of Al_2_O_3_ content, respectively, in CS: AgNO_3_. The measurements were taken at ambient temperature [35]. Copyright: . However, the nanocomposites display frequency-independent behavior at higher frequencies (**A**. *a*). Therefore, ε′ is relatively constant with changes in the frequency; this is because of such rapid field alteration that the dipoles will no more be able to orient themselves in the field direction after effecting in the permittivity and dielectric loss [24]. The rise in dielectric constant in the lower frequency range is mostly due to polarization, which weakens the electrostatic binding strength near grain boundaries. It is worthy to note that the loss of electrostatic binding strength is predominantly affected by polarization [40] © 2019 The Authors. Published by Elsevier B.V. This is an open-access article under the CC BY license (http://creativecommons.org/licenses/BY/4.0/). **B** The scattering and absorption behavior of the dielectric permittivity and tangent δ (**C**) of the Ecoflex®:SWCN hybrid layer vary with different concentrations of SWCN [36].Copyright: © 2021 by the authors. Licensee MDPI, Basel, Switzerland. This article is an open-access article distributed under the terms and conditions of the Creative Commons Attribution (CC BY) license (https://creativecommons.org/licenses/by/4.0/).Outstanding values of the permittivity and ε′ in the low-frequency range (< 1 kHz) are explained by the presence of the extrinsic polarization impact (space charge effect) due to blocking of charge carriers (dipoles) near the electrodes at the sample-electrode interface (named electrode polarization phenomenon) or at boundaries of nanomaterials and polymer matrix of composites [37]. Polarization is defined as the smallest displacement of these charges, which produce dipoles at the material borders. [38]. As known, the ε′ of a substance measures the storing electrical energy ability during exposing time to an electric field, associated with the amount of polarization inside the materials [28]. With frequency increasing, lower values of ε′ could be associated with the polarization relaxation process (dipolar relaxation) [37, 39]

**Fig. 2:**
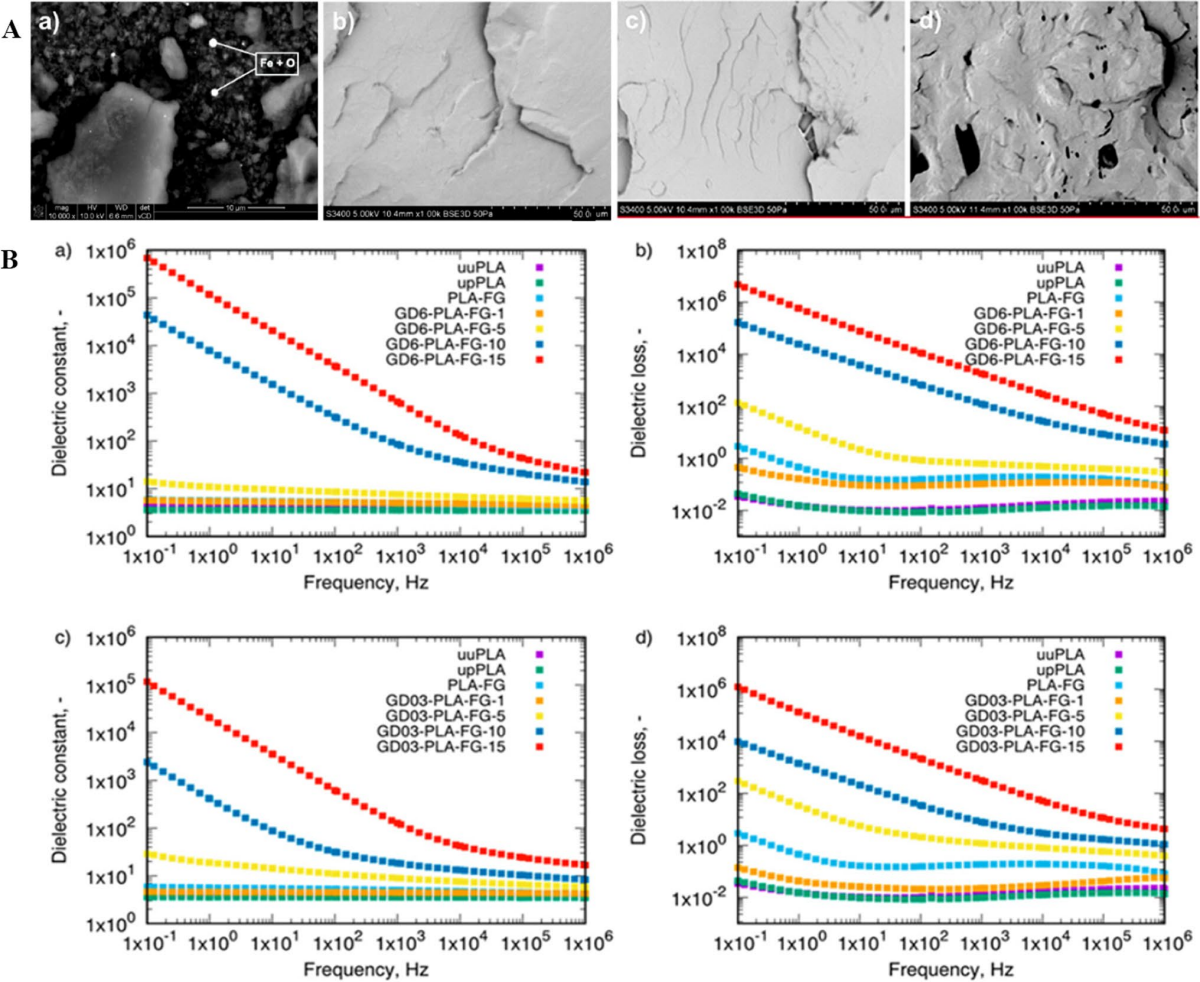
**A** Scanning electron microscope (SEM) images of: (*a*) GD nanoparticles with 6% ash content weight; magnification of 10,000. Fractured surface of (*b *) uuPLA, (*c*) upPLA, and (*d*) PLA-FG; magnification of 1000 × . uuPLA refers to unfilled and unprocessed PLA, upPLA refers to unfilled and processed PLA, and PLA-FG refers to PLA with plasticizer and compatibilizer. **B** (*a*) The dielectric constant of GD6-PLA-FG nanocomposites, (b) The dielectric loss of GD6-PLA-FG nanocomposites, (**c**) The dielectric constant of GD03-PLA-FG nanocomposite, and (*d*) The dielectric loss of GD03-PLA-FG nanocomposites at a temperature of 298.15 K. uuPLA refers to unfilled and unprocessed PLA, upPLA refers to unfilled and processed PLA, PLA-FG refers to PLA with plasticizer and compatibilizer, GD6 refers to graphite/diamond mixture with 6% ash content weight, GD03 refers to graphite/diamond mixture with 0.3% ash content weight, numbers indicate the weight percentage of the filler (GD6, GD03).[46]. Copyright: © 2021 by the authors. Licensee MDPI, Basel, Switzerland. This article is an open-access article distributed under the terms and conditions of the Creative Commons Attribution (CC BY) license (https:// creativecommons.org/licenses/by/4.0/).

**Fig. 3:**
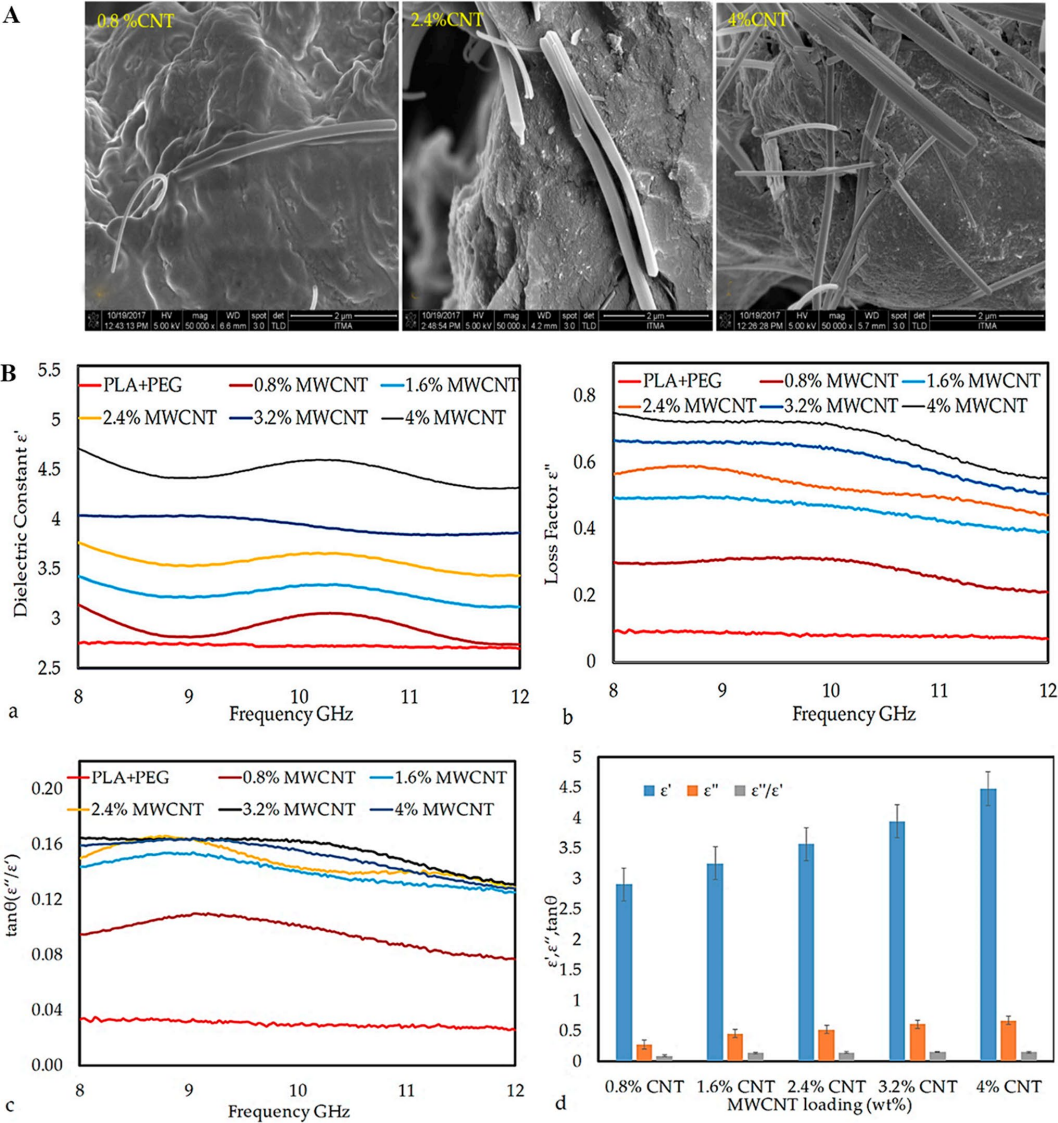
**A** The composite films with varying amounts of multi-walled carbon nanotubes (MWCNT) were analyzed using a Field Emission Scanning Electron Microscope (FE-SEM) to obtain micrographs. **B** Additionally, the frequency dependency of the dielectric properties (*a*) ε′, (*b*) ε″, and c)tan δ were plotted, and (*d*) a comparison of these properties as a function of MWCNT nanoparticle loading was made in MWCNT/PLA/PEG nanocomposites. [32]. Copyright: © 2020 by the authors. Licensee MDPI, Basel, Switzerland. This article is an open-access article distributed under the terms and conditions of the Creative Commons Attribution (CC BY) license (http://creativecommons.org/licenses/by/4.0/)

The most effective method of measuring a particular dielectric characteristic depends on the physical and electrical properties of the material, the required level of accuracy, and the frequency of the measurement. Several instruments can provide reliable measurements of the electrical parameters of an unknown material within a specific frequency range. The Alpha Dielectric Analyzer measures the dielectric properties of BPNC films across frequencies and temperatures and analyzes charge transport and molecular dynamics. Samples coated with silver or gold on both sides act as electrodes or are placed between two gold-plated parallel plate capacitor electrodes [24, 28]. Dielectric relaxation spectroscopy (DRS), broadband dielectric spectroscopy (BDS), impedance analyzer, and rectangular waveguide adapter technique are employed for the purpose of quantifying the dielectric characteristics [29–32]. The determination for the resistance of the BPNCs to an electric field is calculated by the complex dielectric permittivity (ε*) shown in Eq. 1, where ε′ is the real part, and ε′′ is the imaginative part. The dielectric constant (ε′) (Fig. 1(A. a, B. a, and C. a), Fig. 2 (B. a, and B. c), and Fig. 3 (B. a)) measures the electrical energy storing capacity. The ε′′ value measures energy dissipation or energy loss per cycle due to the Joule heating effect, generating heat through dissipating energy (Fig. 1 (A. b, B. b, and C. b) and Fig. 3 (B. b)) [33, 34]. The dielectric constant (ε′) was determined using the Eq. 2 [34]. The loss tangent (tan δ) calculated using Eq. 3 that is commonly used to describe dielectric losses (Fig. 1 (B. c) and Fig. 3 (B. c)) [32].

1 $$\varepsilon * = \varepsilon ^{\prime} - {\text{i}}\varepsilon ^{\prime \prime}$$

2 $$\varepsilon^{\prime} = {\text{C}}_{{\text{p}}} {\text{t}}/\varepsilon_{0} {\text{A}}$$

3 $${\text{Tan }}\delta = \frac{\varepsilon ^{\prime \prime} }{{\varepsilon^{ \prime} }}$$

where C_p_ represents the capacitance, t denotes the thickness of the film, ε_0_ signifies the permittivity of free space, and A is the area of the film.

The frequency dependence of the dielectric constant (ε′ value) can characterize the interfacial polarization (IP) in the low-frequency range. Interfacial polarization (IP) happens when different electrically charged materials create a charge difference at their interface. This occurs in composite materials with two different dielectric mediums, based on the Maxwell–Wagner-Siller's (MWS) IP and Koop's theory [24]. This mechanism is related to the conduction relaxation process, which occurs when randomly oriented charges move by hopping inside the polymer matrix [41]. In contrast, in the higher frequency region, only electronic polarization (molecular and dipolar polarization of the polymers in the presence of nanofiller) [42] dominates, and accumulated charge polarization becomes negligible. Therefore, the dielectric constant decreases [22].

The dielectric losses or dissipation factors (ε″ and tanδ values) of the BPNCs are high in the low-frequency region, due to the presence of all types of polarization. A relaxation peak in the tanδ spectra (Fig. 1(B. c)) is clearly related to the interfacial polarization (IP) relaxation process in the system, and the evidence of charge transfer shows the local flexibility of the polymer chains [43–45].

The dielectric loss values are higher when the material has a conducting constituent (Fig. 2B. b and B. d), which increases the mobile charge carriers that move between the parallel electrodes in the applied alternating electric field. This enhances the total conductivity of the samples, and thus increases the dielectric loss [24]. The dielectric losses decrease in the high-frequency range, as fewer types of polarization occur. A low dielectric loss at higher frequencies is another important parameter [22]. The dielectric loss tangent measures the ratio of electrical energy that a material dissipates to the energy that it stores in a cyclic field [24, 30, 45].

The ε′ and ε″ values of the BPNC increase with the temperature, showing the thermally activated dielectric behavior of a BPNC. As the temperature rises, the polymer matrix creates more free space, which makes the dipolar re-orientation easier and increases the polarization and dielectric permittivity values of the polymer [27]. Consequently, The ε′, ε″, and tanδ values of the BPNC strongly depend on the frequency of the electric field, the amount of nanofiller in the BPNC, and the temperature of the BPNC [42]. Dhatarwal and Sengwa studied the dielectric spectra of poly (vinylpyrrolidone)/poly (ethylene oxide) (PVP/PEO)/5 wt% Al_2_O_3_ nanocomposites and divided them into three separate regions. The 20 Hz to about 1 kHz range is the lower audio frequency (LAF) region, where the ε′, ε″ and tanδ values of the BPNC decrease rapidly with increasing frequency. The 1 to 20 kHz range is the higher audio frequency (HAF) region, where the values decrease slowly. The 20 kHz to 1 MHz range is the radio frequency (RF) region, where the ε′ values are almost independent of frequency [42].

### Effect of nanofillers on dielectric properties

The dielectric behavior of BPNCs depends on the type of nanofillers and their interaction with the polymer matrices [21, 47]. In general, the chemical structure and polarization of BPNCs affect their dielectric properties [23]. Xu et al. performed several dielectric spectroscopy measurements to compare how silica and clay nanoparticles affect the nanocomposites. They found a new and slower relaxation mode in the clay nanocomposite, which did not interfere with the local relaxation motion of the poly(lactic-co-glycolic acid) (PLG) chains. They suggested that the clay nanoparticles and the PLG chains adsorbed at the interface created physical crosslinks, which slowed down the relaxation of those PLG chains [47]. Moreover, poly (vinyl alcohol) (PVA) has a high dielectric constant in the low frequency region when it contains CuO nanofillers. This is because the CuO nanofillers create IP in the PVA. The dielectric loss of the CuO BPNC is almost unchanged, which makes it a good insulating material [48].

The dielectric permittivity of BPNCs depends on the frequency and how it affects the polarization dipole moment and electrical conductivity. For example, the dielectric properties of the polymer-based matrix increased when more conductive multi-walled carbon nanotube (MWCNTs) fillers were added (Table 1). The weight percentages of MWCNTs increased gradually [32]. The presence of two-dimensional MoS_2_ nanosheets in the polymeric matrix increased its dielectric properties and IP due to their specific surface area and high charge polarization strength. As more nanosheets were added, the dielectric constant increased and the dielectric loss decreased, due to the stronger interaction between the nanosheets and the matrix under an external electric field [31].

**Table 1:** Demonstrates various types of BPNCs, with nanofillers and biodegradable polymer matrices and their applications

Polymer	Nanofiller	Application	References
Polyvinyl alcohol (PVA)	CuO nano ellipsoids	Dielectric material in microchips, suspension insulators etc	[48]
Polylactic acid (PLA)	Core-shell BaTiO_3_ nanoparticles	Environmentally friendly high-performance energy storage devices	[28]
Cellulose/Polyaniline	Cobalt ferrite nanoparticles	Electromagnetic, antimicrobial	[24]
Chitosan/polyacrylic acid/polypyrrole/	Silver nanoparticles	Conducting bionanocomposites hydrogel	[29]
Polyvinyl alcohol (PVA)	CaCO_3_ nanoparticles	Biodegradable packaging	[25]
Chitin	Molybdenum disulfide (MoS_2_) nanosheets	High-performance biomass-based dielectric capacitors	[31]
Poly (vinyl pyrrolidone) (PVP)	Tin oxide (SnO_2_)	Flexible-type advanced electronic devices	[44]
Poly (vinyl pyrrolidone) (PVP)	Zinc oxide (ZnO) and titanium dioxide (TiO_2_) nanoparticles	Eco-friendly organoelectronic devices	[34]
Poly (vinyl chloride) (PVC)	Nano-SiO_2_	Electronic components packaging	[43]
Chitosan	2-D graphene oxide	Optoelectronic or electrical devices operating at high frequencies	[20]
Polycaprolactone (PCL)	Graphene oxide	Osteogenic and drug-eluting scaffolds	[49]
Poly (vinyl pyrrolidone)/poly (ethylene oxide) PVP/PEO	SnO_2_ nanoparticles	Multifunctional advanced materials	[34]
Chitosan	Silver nanoparticles	UV and electromagnetic shielding materials	[26]
Poly (vinyl pyrrolidone)/poly (ethylene glycol) PVP/PEO	Alumina (Al_2_O_3_) nanoparticle	Nanodielectrics eco-friendly flexible device	[42]
Chitosan/polyaniline	Calcium copper titanate (CCTO) and reduced graphene oxide (rGO)	Electronic devices and high-frequency applications	[30]
Poly (vinyl alcohol) (PVA) and poly(vinyl pyrrolidone) (PVP) blend	Amorphous silica (SiO_2_) nanoparticles	Flexible nanodielectric	[27]
polyvinyl acetate (PVAc)	Cellulose nanocrystals (CNC)	Separators for ionic battery	[50]
Polylactide (PLA)	Titanium dioxide decorated multi-walled carbon nanotubes (MWCNTs@TiO_2_)	Insulating layers to effectively uppress the dielectric loss	[51]
Cellulose	Boron nitride nanosheet (BNNS)	Dielectric energy storage devices	[52]
Poly lactide (PLA) and poly (butylene adipate-co-terephthalate) (PBAT)	Graphene nanoplatelets (GNPs)	Electromagnetic interference shielding material	[53]

The breakdown strength is a crucial factor that needs to be considered during the practical implementation of BPNCs. It establishes how to determine the maximum electric field and energy storage density of BPNCs. Two factors affect their breakdown strength: interface strength and nanoparticle dispersion [28]. For instance, adding nano-BaTiO_3_ to a poly(lactic acid) (PLA) matrix improved the breakdown strength due to better adhesion [28].

Muthupandeeswari et al. found that adding CaCO_3_ nanofiller to poly(vinyl alcohol) (PVA) BPNCs lowered ε′ value, reducing conductivity. This makes it suitable for microelectronic packaging and optoelectronic devices [25]. The system has more charge carrier concentrations and higher ε′ values and conductivity when it contains nanofillers. However, if the nanofiller concentration is too high, the ε′ value may decrease. This is because the nanofillers may clump together in the polymer matrix and reduce the number of charge carriers [45]. Deshmukh et al.'s study demonstrated that the tanδ and ε′ values increased when the PVA and Poly(vinyl pyrrolidone) (PVP) blend films had more SiO_2_ polar nanofillers. This was because the SiO_2_ nanofillers interacted with the polar groups of the PVA and PVP [23]. The addition of TiO_2_ and ZnO nanoparticles increased the ε′ values of PVP due to electrostatic interactions with polar groups, making BPNC suitable for energy storage applications [34]. The study by Dhatarwal, Choudhary, et al. also showed that the dielectric permittivity of the poly (vinyl pyrrolidone)/ SnO_2_ BPNCs could be increased and adjusted by changing the concentration of the SnO_2_ nanoparticles [44].

### Recent advances in dielectric properties of biodegradable polymer nanocomposites

The chemical structure and polarization of BPNCs affect their dielectric properties. Figure 2A a–d and 3A show the SEM morphology images of some BPNCs. The dielectric nanocomposites have a high dielectric constant and a high dielectric loss at the low-frequency region. This makes them suitable for flexible organic electronics that can store charge effectively [5]. Dielectric loss spectroscopy (ε″) identifies BPNCs relaxation mode and interaction with nanofillers and matrices [31]. Nanofillers trapped in the polymer matrix cause IP in BPNCs, leading to higher dielectric constant (ε') in lower frequency region. The nanofillers can improve the dielectric constant without increasing the dielectric loss. The BPNCs with a low dielectric loss, are good insulating materials ^29^. The BPNC has higher ε′, ε″, and tan δ values when it contains conductive MWCNTs fillers. This is because the MWCNTs form a conductive material in the BPNC. The loss factor of the dielectric properties measures the conductivity of the BPNC. The conductivity is important for the absorption and attenuation of radiation (Fig. 3(B. a and B. b)) [32].

The matrix conductivity decreased when the ε′ value was low. This made the BPNCs useful for biodegradable packaging, microelectronic packaging, and optoelectronic devices. They could replace the non-biodegradable packaging materials [9].

**Fig. 4:**
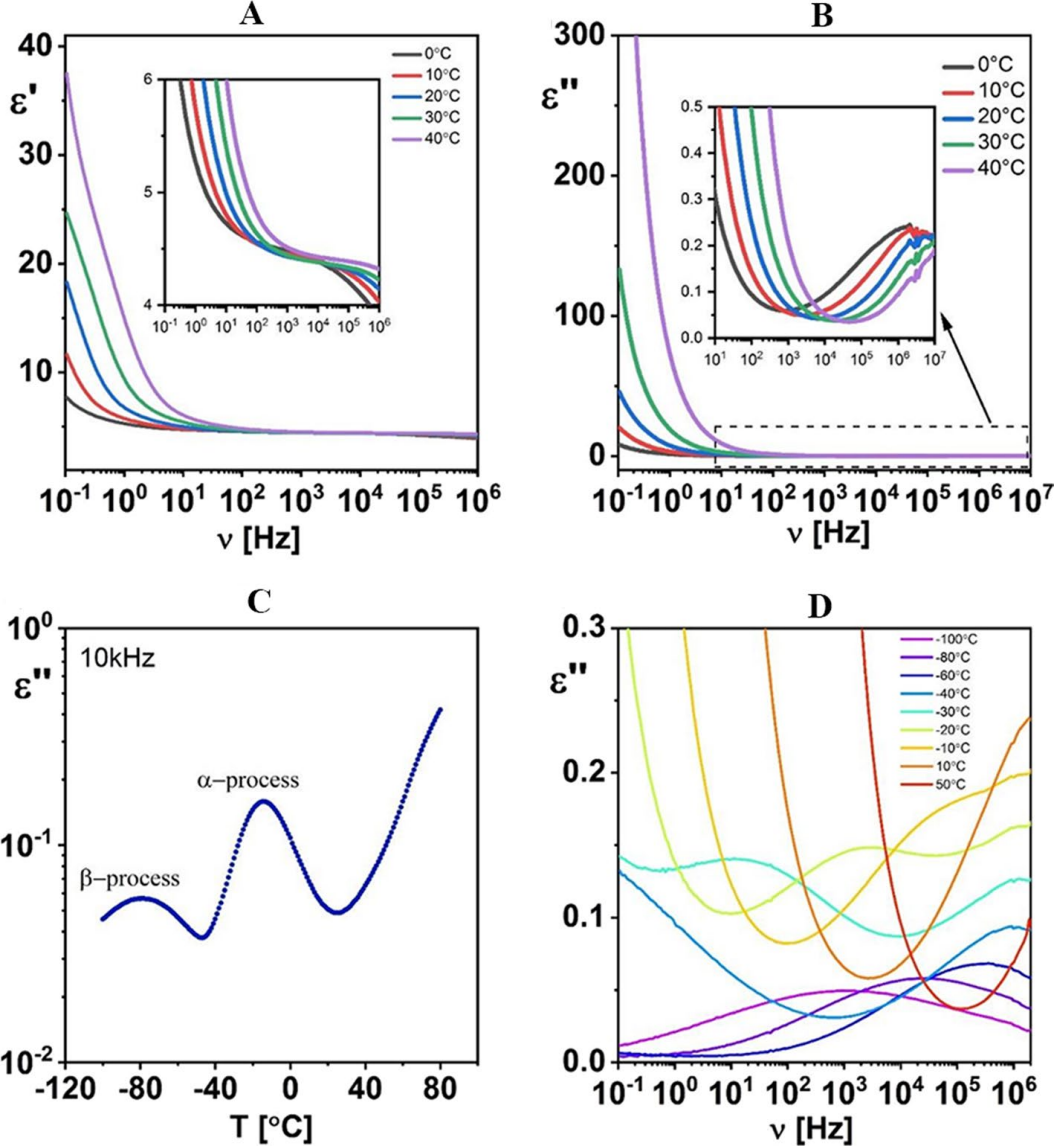
Measurement of the temperature effects on the dielectric properties including dispersion (**A**) and absorption (**B**), as well as the dielectric absorption at 10 kHz (**C**) and specific selected temperatures (**D**) for pure Ecoflex® la yer. [36].Copyright: © 2021 by the authors. Licensee MDPI, Basel, Switzerland. This article is an open-access article distributed under the terms and conditions of the Creative Commons Attribution (CC BY) license (https://creativecommons.org/licenses/by/4.0/). Biodegradable and renewable eco-friendly polymers have several advantages: light-weighted, excellent mechanical properties, flexibility, good processability, and low dielectric loss [28, 31]. Traditional ceramic materials have advantages such as a high dielectric constant and minimal dielectric loss; however, they often possess poor breakdown strength and flexibility [28]. Along with the benefits of biodegradable polymers, they display low dielectric permittivity at room temperature. Therefore, to achieve the optimum dielectric values of polymer materials, there is a demand for uniform diffusion of a suitable nanofiller in an appropriate low content, known as polymer nanodielectrics (PNDs), so that it does not interfere with PNDs’s technological performance and without agglomeration in the polymer matrix following the intended application [42]. The nanofiller concentration-dependent dielectric permittivity, and sensibly low values of the tailored dielectric BPNCs, indorsed their application as tunable next-generation polymer nanodielectrics, with multifunctional properties, including flexible-type biodegradable microelectronic components, energy-storing capacitors, and various technological and industrial applications [14]. Also, the low dielectric constant and low dielectric loss materials are appropriate for insulator purposes in microelectronic devices [27]

The BPNC film has dielectric properties (ε′, ε″, and tan δ) that can be adjusted. These properties show that the BPNCs are suitable for nanodielectrics in biodegradable electronics [36]. The BPNC has higher ε′ and ε″ values when the temperature increases. This shows that the BPNC has a dielectric behavior that depends on the temperature (Fig. 1Ca-c) and Fig. 4 A-D) [36]. The elevated temperature induces an increase in the available voids within the polymer matrix, which makes it easier for the dipoles to re-orient. This increases the polarization and the dielectric permittivity of the polymer [9].

## Electrical conductivity of biodegradable polymer nanocomposites

### History

Conductive BPNCs refer to innovative polymers that possess strong π-conjugation along with magnetic, electronic, and optical properties similar to metals. The inclusion of nanoscale particles in the polymer matrix enhances their conductivity. These conductive BPNCs find extensive applicability in various industries and medical fields. They are utilized for coating and preventing corrosion, as well as in the development of sensors, catalysts, energy storage and conversion systems (such as batteries, supercapacitors, dielectric capacitors, and solar cells), biomedical devices, tissue engineering, anti-static agents, and optical and electroactive devices [43, 45, 54, 55]. Conjugated polymers have been studied since the early 1970s, when sulfur poly-nitride [(–S=N–) x] was found to have a metallic-like conductivity (~ 103 S/cm) at room temperature. This remarkable property sparked the interest of the scientific community, which searched for other compounds with similar properties. Conjugated biocompatible polymers are promising material for developing effective, convenient, and reliable implants. Implantable biomedical electrodes are extensively utilized to record and stimulate biological signals, encompassing a broad spectrum of applications ranging from the treatment of Parkinson's disease to the facilitation of brain-machine interfaces. One of the main challenges that scientists face in designing bioelectronic implants is how to interface rigid electronic devices with the soft tissue in our bodies. The problem affects the device performance, as solid electronic devices communicate through current, while biological tissues communicate through ions. This review article provides a comprehensive overview of the conductivity of BPNCs, which have been extensively investigated for their multiple advantages. Polymers have low conductivity because of the complex arrangement of polymer chains and the lack of free electrons in their systems. This limits their range of potential applications. Therefore, incorporating nanoparticles into polymer structures has been proposed to enhance their conductivity [56].

### Fabrication of conductive biodegradable polymer nanocomposites

In order to induce electrical properties in biodegradable polymer nanocomposites, conductive polymers like polypyrrole, polyaniline, and polythiophene can be utilized. However, these synthetic polymers are non-biodegradable, which is a major challenge in solving environmental problems [57–62]. Therefore, this review article focuses on recent advances in biodegradable polymer development as a more environmentally friendly alternative. Conductive BPNCs have potential applications in the biomedical field for neural tissue regeneration, smart implants, and electro-stimulated drug delivery systems [57] (as shown in Fig. 9). Various techniques are available for fabricating BPNCs and incorporating conductive nanoparticles into the polymer matrix, including ex-situ (sequestered synthesis), in-situ (sequential synthesis), and single-pot (concurrent synthesis). These methods have different advantages and disadvantages. Although the ex-situ method is easy to process, it has limited applications. The in-situ method allows more control over the parameters; however, it is more complicated. The single-pot method is faster than the other methods. Nevertheless, it has less control over the structure and morphology of the products [45].

### Percolation theory and excluded volume theory of percolation

Several studies have demonstrated that the conductivity of polymers can be improved by incorporating nanocomposites. Two major theories explain the theoretical and experimental results of this process: the "Excluded Volume Theory of Percolation" and the "Percolation Theory" [47]. The *Excluded Volume Theory of Percolation* explains how the volume of an object prevents other objects from occupying the same space in the system. The Percolation Theory elucidates the mechanism by which nanoparticles induce a transition in the polymer's electrical properties, transforming it from an insulator to a conductor when they reach a critical concentration [48]. The Theory of Percolation elucidates the concept of the percolation threshold, denoting the minimal concentration of nanoparticles requisite for inducing conductivity in the polymer. The electrical conductivity of BPNCs is contingent upon the geometric parameters of the filler particles, including their size, shape, and aspect ratio (defined as the ratio of length to diameter) [43]. The primary determinant impacting the percolation threshold of BPNCs is the aspect ratio of the filler. The percolation threshold exhibits an inverse relationship with the aspect ratio, signifying that it rises as the aspect ratio diminishes. This observation aligns with both experimental and theoretical findings concerning isotropic networks [48]. Nanoparticles with large aspect ratios can lower the percolation threshold of polymer composites. This implies that a reduced quantity of nanoparticles is required to confer conductivity to the polymer [47]. In addition to the percolation threshold, the electrical conductivity of the BPNCs is also influenced by the orientation of the nanofillers. The nanofillers may orient themselves in various directions within the polymer matrix, altering their electrical conductivity of the BPNCs [50]. Carbon nanotubes in multi-walled nanotubes have the highest conductivity when they are aligned at 30°. However, they have the lowest conductivity when they are aligned at 0° [47]. The addition of doped nanoparticles to the polymer matrix alters the electrical and optical properties of polymer composites by introducing free charge carriers. Doping can manifest as either. P-type doping involves the extraction of an electron from the highest occupied molecular orbital (HOMO) of the polymer, thereby augmenting the hole carrier density. Conversely, n-type doping entails the introduction of an electron into the lowest unoccupied molecular orbital (LUMO) of the polymer, leading to an augmentation in the density of electron carriers. Conjugated polymers can conduct both electrons and ions, unlike other polymers such as polystyrene (PS) or Nylon. The rationale behind this phenomenon is that conjugated polymers possess a molecular backbone characterized by alternating single and double bonds, thereby facilitating the unrestricted movement of electrons. When they are in an aqueous environment, the flow of electrons is balanced by the flow of ions. However, conjugated polymers can also form complex shapes and patterns, like other polymers. This is important for making electronic implants that are compatible with biological tissue [63]. An impedance analyzer measures the electrical conductivity of BPNCs by cutting samples into small pieces and measuring their electrical properties at different temperatures and frequencies. The direct current (DC) electrical conductivity of BPNCs is measured using the four-probe technique, which involves a DC or an alternating current (AC) source and a nanovoltmeter [64].

### Effect of nanofiller dispersion on the bpncs electrical conductivity

**Fig. 5:**
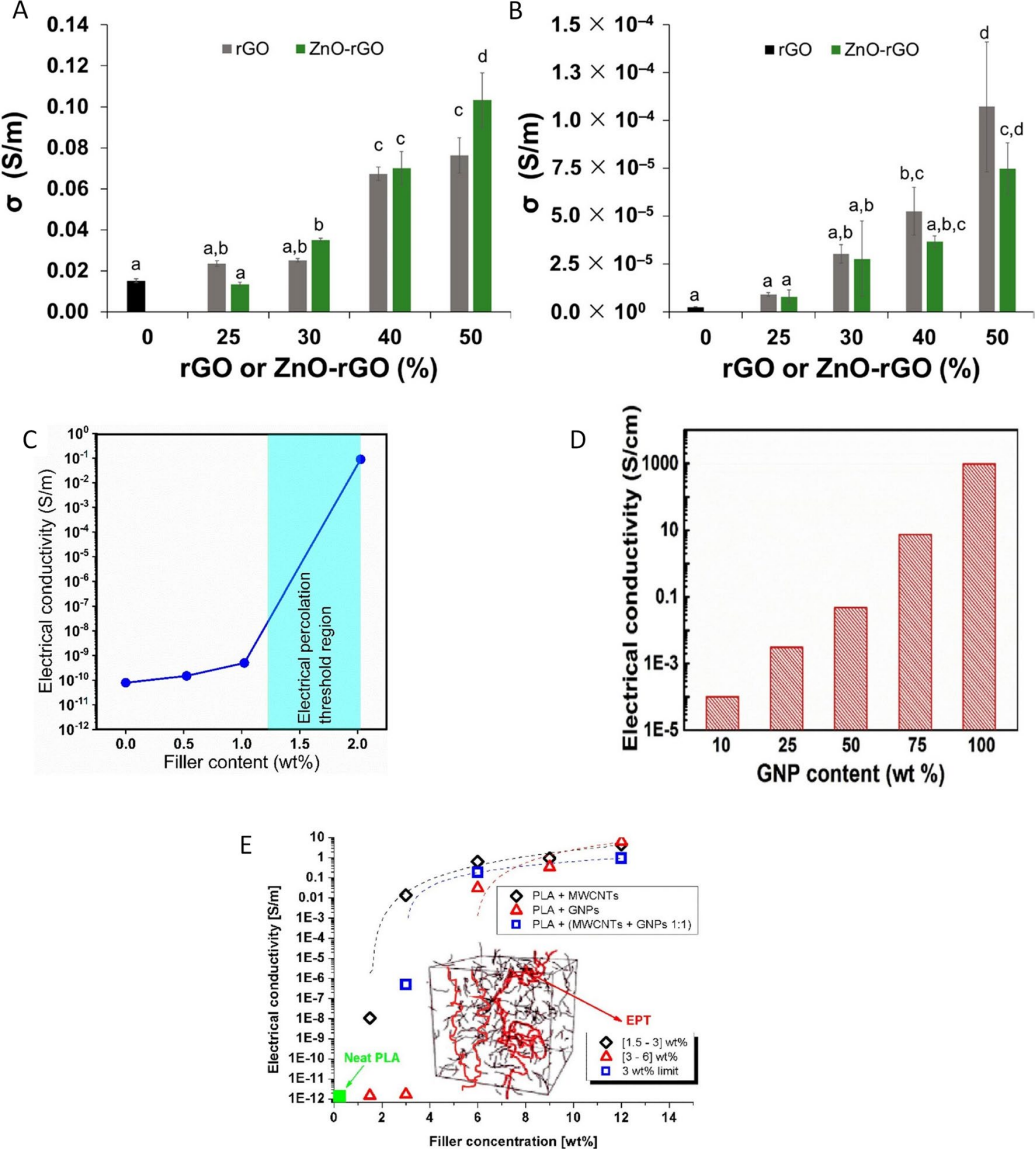
The electrical conductivity of alginate and bionanocomposites with varying nanoparticle: in-plane (**A**) and through-plane (**B**) [66]. Copyright: © 2021 by the authors. Licensee MDPI, Basel, Switzerland. This article is an open-access article distributed under the terms and conditions of the Creative Commons Attribution (CC BY) license (https://creativecommons.org/licenses/by/4.0/). *Effect of Nanofiller Loading on BPNCs Electrical Conductivity.* **C** The electrical conductivity of nanocomposites made of PP/PLA40 and MWCNTs tested at various levels of filler content. **D** The amount of GNPs in GNP/NFC composite paper was varied to measure its electrical conductivity. **E** The relationship between electrical conductivity and the percentage of filler content, examined in different systems. The inset of the study shows a schematic representation of electric percolation thresholds (EPT) and the values obtained in this research. [71]. Copyrights: © 2019 by the authors. Licensee MDPI, Basel, Switzerland. This article is an open-access article distributed under the terms and conditions of the Creative Commons Attribution (CC BY) license (http://creativecommons.org/licenses/by/4.0/). © 2019 by the authors. Licensee MDPI, Basel, Switzerland. This article is an open-access article distributed under the terms and conditions of the Creative Commons Attribution (CC BY) license (https://creativecommons.org/licenses/by/4.0/). © 2019 by the authors. Licensee MDPI, Basel, Switzerland. This article is an open-access article distributed under the terms and conditions of the Creative Commons Attribution (CC BY) license (http://creativecommons.org/licenses/by/4.0/)

Previous studies of biopolymer nanocomposites have shown that nanoparticles should be uniformly dispersed within the polymer matrices. Furthermore, the conductivity of BPNCs increases with better nanoparticle dispersion in the polymer matrix (Fig. 5A and B). This phenomenon can be ascribed to the improved interfacial load transfer [51]. However, the strong inter-van der Waals forces of nanofillers increase their surface energy, making them tend to agglomerate in polymer matrices. To solve this problem, surface functionalization is often used to achieve uniform distributions and improve the compatibility of polymer matrices [52]. Outstandingly, the shape of the nanoparticles affects the percolation threshold and conductivity of nanocomposites. For spherical nanoparticles, the percolation threshold is directly proportional to the size of nanofillers, which increases with the size of nanoparticles [53], albeit, for cylindrical and layered particles, the percolation threshold demonstrates an upward trend as the aspect ratio decreases [65]. S. Kashi et al. found that pure PBAT had higher electrical conductivity (0.11 S/m) than pure PLA (0.02 S/m). However, PLA nanocomposites with 9–15 wt% Graphene Nanoparticles (GNPs) had higher conductivity than PBAT nanocomposites. Increasing the nanofiller quantity to 15 wt% resulted in a significant increase in the conductivity of PLA nanocomposites to 7.4 S/m, which was 2.5 times greater than that of PBAT nanocomposites. The AC and DC conductivity values of these biopolymer-based nanocomposites followed similar trends [53].

The DC and AC conductivity of BPNCs are significantly affected by the nanofiller loading (Fig. 5C–E). A recent study on PVA/Starch/Graphene nanocomposite and their electrical conductivity showed that the conductivity increased dramatically after adding nanoparticle loading. They explained this high AC and DC conductivity by the percolation threshold effects of BPNCs. The main challenge in the commercialization of carbon nanotubes and graphene is the possibility of aggregation during the processing of such BPNCs [67]; this phenomenon stems from solid van der Waals forces of their intricate entanglement and unique sp^2^ bonding [47]. It has been reported that increasing particle loading also increases the tendency for aggregation. It can be inferred that the uniform distribution of nanofillers within polymer matrices has the potential to significantly augment the conductivity of the PNs. However, some researchers have shown that using aggregating particles to form conductive networks can also improve the electrical properties of BPNCs, because of the effective physical contact between the nanoparticles [53]. Kim et al. [68] studied Epoxy/Graphene nanoplatelets nanocomposites and found that well-dispersed or exfoliated graphene nanoplatelets (GNPs) had electrical resistivity two orders higher than poorly-dispersed or aggregated GNPs. In a recent study, nanocomposites with dispersing GNPs in PLA and PBAT were fabricated. They reported that PLA had relatively poor distribution of graphene particles above the percolation threshold (5%wt), but PBAT/GNP nanocomposites had lower electrical conductivity. The paradoxical observation was elucidated by attributing it to the development of a co-continuous structure comprising graphene-rich and graphene-poor phases within the novel PBAT/graphene nanocomposites. Furthermore, it was posited that the enhanced conductivity of the PLA nanocomposite primarily stemmed from interfacial polarization, arising from the migration of charge carriers across the interfaces [53]. Aguilar et al. conducted a study to examine the impact of carbon nanotube (CNT) clustering on the electrical conductivity of polysulfone. Their findings revealed that composites containing uniformly dispersed nanotubes exhibited a higher percolation threshold compared to composites with agglomerated CNTs. Interestingly, specimens with well-dispersed CNTs demonstrated significantly lower conductivity than those with agglomerated nanotubes, with notable disparities ranging from 4 to 2 orders of magnitude for CNT ratios exceeding the percolation threshold. [69]. In recent decades, engineers and scientists have tried to modify the optical, electrical, and processing properties of biopolymers, especially their solubility and biocompatibility [55]. According to a study by Nezakati et al. [70], graphene improved the mechanical stability and electrical conductivity of polyhedral oligomeric silsesquioxane polycaprolactone biopolymer.

### Effect of temperature on the BPNC's electrical conductivity

The electrical conductivity of PVP/PVA/CdCl_2_ composite with different loading of CdCl_2_ as a function of temperature has been investigated by Pandey et al. [63]; The conductivity of PVA/CdCl2 electrolyte depends on the temperature. At higher temperatures, it acts as a conductor, but at lower temperatures, PVA/PVP/CdCl_2_ has the highest conductivity. This is because of the regular and accurate arrangement of polymer chains that facilitates the electron flow rate. The highest DC conductivity is achieved by a single polymer PVA with 10% CdCl_2_, which is directly related to the polymer chain density that hinders the electron flow rate in a double polymer system. The effect of temperature shows that increasing the temperature makes the polymer chain more likely to rearrange, which helps the electron flow rate. However, if the temperature exceeds a certain level, the conductivity will decrease. In another study conducted by Jahan [72], the DC electrical conductivity of biopolymer gellan gel -a water-soluble anionic polysaccharide- electrolytes was studied The results demonstrated that the conductivity of gellan gel is contingent on the temperature and increased with higher temperature, as expected. This behavior was similar to that of a semiconductor. The conductivity of gellan gel electrolytes also increased with different polymer concentrations at higher temperature. For a polymer concentration of Cp = 0.5 wt%, the conductivity increased non-linearly, while for a polymer concentration of Cp = 2 wt%, the conductivity increased linearly with temperature. This phenomenon was related to the change in their conformation [63].

### Effect of matrix properties on the BPNC’s electrical conductivity

The percolation threshold is influenced by the matrix properties, including polarity, degree of polarization, and viscosity. Previous studies have shown that the conductivity is similar for the same matrices with the same nanoparticle concentration. However, the magnitude of variation among different matrices is subject to a substantial disparity of ten or more orders. The interactions between nanoparticles also affect the formation of conductive networks in BPNCs, as the surface chemistry of the nanofillers and the chain conformation determine the conductivity of PNs. Another factor to consider is the interphase, which is a distinct phase in BPNCs. This phase is generated by the substantial surface area of the fillers, resulting in a persistent interfacial interaction between the fillers and the polymer matrix. It has been documented that the percolation threshold exhibits an inverse relationship with the weighted average of the length distribution [73]. Scientific research has demonstrated that the incorporation of minute quantities of elongated rods can considerably diminish the percolation threshold. This phenomenon becomes increasingly conspicuous as the number of elongated rods within the system increases [74]. The inverse relationship between the percolation threshold and the quantity of conductive nanoparticles suggests that the electrical conductivity of the nanocomposite is contingent solely upon the existence of conductive fillers, rather than the polymer matrix [75].

### Recent advances in the electrical conductivity of biodegradable polymer nanocomposites

Some biodegradable polymers, such as polyaniline or polythiophene, have inherent electrical conductivity. They can be mixed with other biodegradable polymers to make composites with improved electrical properties. Natural materials, like cellulose [76, 77] or chitin, can also be used as reinforcing agents in biodegradable polymer composites to increase their electrical conductivity [63]. Biopolymer-based nanocomposites with good electrical conductivity are needed to reduce the polymeric pollution caused by electronic wastes. However, a major challenge for these nanocomposites is to achieve the desired physical and mechanical properties. To do this, biopolymers are often loaded with various nanoparticles. The optimal enhancement of the electrical properties of biopolymers depends on the amount and shape of the nanoparticles, as well as the temperature. Natural nanoparticles have been widely used in the past, but their optimal amount was sometimes hard to determine. Nowadays, biodegradable nanoparticles, either natural or synthetic, are used to fabricate biopolymers with suitable electrical properties. As mentioned earlier, effective electrical conductivity can be attained by controlling the nanofiller parameters and the temperature.

## Thermal behavior of biodegradable polymer nanocomposites

As mentioned before, some atomic defects and random orientation, impurities, vacancies, voids, and polymer chain entanglement hinder the thermal conductivity of polymer [78]. To meet the needs in this regard, inorganic nanoparticles possessing elevated thermal conductivities are employed as additives to enhance the thermal characteristics of biopolymers. Prior investigations have demonstrated a significant correlation between the thermal conductivity of biopolymer nanocomposites (BPNCs) and the concentration as well as dispersion of the nanoparticles [74, 79, 80]. Therefore, synthetic elements such as zero-dimensional (0D) nanoparticles, one-dimensional (1D) nanofibers/nanorods/nanotubes, or two-dimensional (2D) nanoplates/nanosheets are introduced into the biopolymer matrix as active nanostructured constituents, thereby engendering novel functionalities. For instance, carbon nanocomponents can be integrated into a biopolymer matrix, leading to the creation of BPNC possessing adjustable thermal and electrical conductivity [81–83].

As previously stated, the thermal conductivity of nanofibers exhibits a decline as their diameters increase, because the fiber surface may restrict the random orientation of polymer chains. The filler–matrix interface also affects the thermal conduction process of nanocomposites [81]. Biopolymers, as the most prevalent form of polymer, finds extensive application across diverse fields. Over the past few decades, numerous scholars have conducted extensive investigations into organic and inorganic nanocomposite materials [84, 85]. For example, the incorporation of electrically conductive additives, such as carbon black, carbon nanotubes, nanofibers, graphene, and Fe_3_O_4_, to biopolymers can enhance their thermal properties and create unique thermal and electrical properties [86]. Within this section, we shall deliberate upon the impact of nanofiller properties, such as dispersion, size, geometry, and loading, influence the thermal conductivity and thermal stability of polymeric nanocomposites.

### Thermal conductivity of BPNCs

**Fig. 6:**
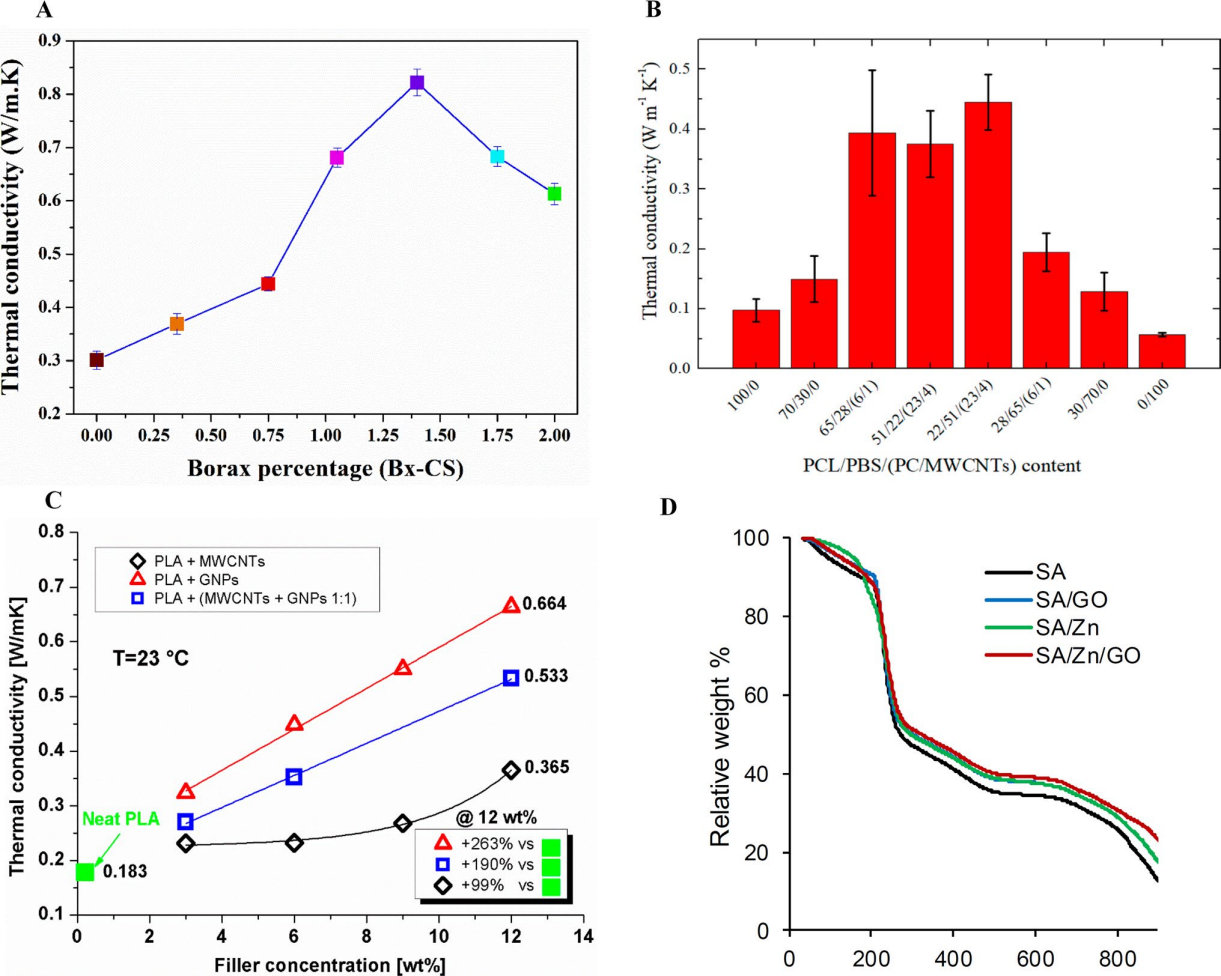
**A** Thermal conductivity of biopolymer films made from cassava starch with varying levels of borax addition [90]. Copyright: © 2021 by the authors. Licensee MDPI, Basel, Switzerland. This article is an open-access article distributed under the terms and conditions of the Creative Commons Attribution (CC BY) license (https:// creativecommons.org/licenses/by/ 4.0/). **B** Effect of PC/MWCNTs masterbatch content on thermal conductivity of PCL/PBS blends and their respective nanocomposites [91]. Copyright: © 2019 by the authors. Licensee MDPI, Basel, Switzerland. This article is an open-access article distributed under the terms and conditions of the Creative Commons Attribution (CC BY) license (http://creativecommons.org/licenses/by/4.0/). Influence of PC/MWCNTs masterbatch content on the thermal conductivities of the nanocomposites. **C** Thermal conductivity variations of CNT/PLA, GNP/PLA, and (CNT + GNP 1:1)/PLA composites with varying filler concentrations [71]. Copyright: © 2019 by the authors. Licensee MDPI, Basel, Switzerland. This article is an open-access article distributed under the terms and conditions of the Creative Commons Attribution (CC BY) license (http://creativecommons.org/licenses/by/4.0/). **D** TGA thermograms results [109]. Copyright: © 2020 by the authors. Licensee MDPI, Basel, Switzerland. This article is an open-access article distributed under the terms and conditions of the Creative Commons Attribution (CC BY) license (http://creativecommons.org/licenses/by/4.0/)

Ceramic nanofillers, including boron nitride (BN), beryllium oxide (BeO), silicon carbide (SiC), aluminum nitride (AlN), silica (SiO_2_), titania (TiO_2_), alumina (Al_2_O_3_), zinc oxide (ZnO), and others, are commonly utilized in the production of BPNCs. However, these fillers are characterized by their high cost and limited suitability for large-scale manufacturing and application. Consequently, researchers are actively seeking alternative lightweight carbon-based fillers, such as carbon nanotubes (CNTs), carbon blacks (CBs), vapor-grown carbon fibers (VGCFs), carbon fibers (CFs), and graphite [87]. Graphene nanoplatelets (GNPs) are also good fillers for making thermally conductive BPNCs [88]. Furthermore, the thermal interface resistance existing between the polymer matrix and fillers poses a constraint on the augmentation of thermal conductivity in BPNCs. This limitation can be mitigated through the implementation of various techniques, including surface modification [89]. In this study, borax was added to cassava starch biopolymer film. The findings indicate that the thermal conductivity exhibited a linear increase in proportion to the borax content, owing to the augmentation of heat transfer. According to the data presented in Fig. 6A, the maximum thermal conductivity was attained when the volume fraction of borax reached 1.40%. However, when the borax volume fraction exceeded 1.4%, the thermal conductivity decreased because of the heat carrier scattering phenomena [90]. In another study, the thermal conductivity of polycaprolactone (PCL)/polybutylene succinate (PBS) blends with weight ratios of 70/30 and 30/70, as well as their respective nanocomposites containing a masterbatch of polycarbonate (PC)/multiwalled carbon nanotubes (MWCNTs), was examined (Fig. 6B). The findings indicated that the inclusion of carbon nanotubes resulted in an augmentation of the thermal conductivity in the blend nanocomposites, which possess a notably high thermal conductivity. However, the 28/65/(6/1) sample had much lower thermal conductivity than the other samples. This could be due to the larger diameter of the dispersed phase particles, which reduced the interactions between them [91]. Figure 6C illustrates the thermal conductivity characteristics of polylactic acid (PLA) infused with varying weight proportions of multi-walled carbon nanotubes (MWCNTs), graphene nanoplatelets (GNPs), or a combination of both fillers (MWCNTs/GNPs). The findings reveal that PLA composites incorporating GNPs as two-dimensional fillers exhibited superior heat conduction compared to the other composite variations. Notably, the highest thermal conductivity value was attained with a concentration of 12 wt% GNPs [71].

#### Effect of filler loading on the thermal conductivity of BPNCs

In general, the thermal conductivity of polymer composites tends to increase in proportion to the quantity of conductive fillers incorporated. However, it is important to note that these composites are unable to attain the intrinsic thermal conductivity values of the fillers themselves [92]. A practical approach to augment the thermal conductivity of polymer composites involves the establishment of a percolated pathway utilizing conductive additives, thereby establishing a network structure embedded within the polymer matrix. The extent of this enhancement predominantly relies on the intrinsic conductivity of these additives [88]. Nevertheless, a substantial filler loading is typically required to establish the aforementioned uninterrupted filler network. This, however, may entail certain disadvantages including subpar processability, compromised mechanical properties, and escalated expenses [93]. Additionally, it has been demonstrated that the thermal conductivity of composites exhibits enhancement solely when the filler content surpasses 30wt%. As an illustration, the thermal conductivity experienced a tenfold increase within the range of 30–50%wt filler content [89, 92]. Therefore, the filler content should be neither too low nor too high to achieve optimal results [89].

#### Effect of filler dispersion on the thermal conductivity of BPNCs

The distribution of filler particles within the polymer matrix plays a crucial role in determining the thermal conductivity of BPNCs. Numerous contemporary investigations have identified it as a fundamental element in augmenting thermal conductivity [88, 92]. Hence, various methodologies, such as functionalization, high-power ultrasonication, and surfactant-enhanced processing, have been suggested to enhance the dispersion of nanofillers within the polymer matrix. For instance, the application of chemical surface treatments, such as silane-based coupling agents, or surface functionalization, can prove advantageous. Besides, it has been observed that a critical mixing time must be exceeded for optimal filler dispersion, and this duration is found to increase proportionally with the filler content [87, 94].

#### Effect of filler geometry on the thermal conductivity of BPNCs

The effective thermal conductivity (keff) of BPNCs is subject to the influence exerted by the shape and dimensions of the filler material. For example, smaller fillers have higher interfacial thermal barrier resistance, which hinders phonon transport and reduces the keff of the composites [94]. Therefore, longer fillers have higher thermal conductivity than shorter ones. Smaller particles also have more extended interfacial regions, which cause phonon scattering and inhibit phonon transport, resulting in lower thermal conductivity [92, 93]. Thus, nanoparticles may not be the optimal choice for achieving high thermal conductivity composites. Moreover, at the same volume, long-fillers have higher thermal conductivity than spherical particles, because they have shorter paths [92].

### Thermal stability of BPNCs

Amorphous metal nanoparticles can be used to reinforce crystalline polymers and create composites with unique thermal properties [95]. The thermal stability of BPNCs can be improved by crosslinking polymer strands with physical means and metal fragments, forming a network of polymer nanoparticles. This network stabilizes the structure by limiting the thermal movement of polymer chains [96]. However, hybrid materials may have lower thermal stability due to the catalytic effect of metals on the oxidative degradation of polymers [87, 89]. Silica nanoparticles can also be incorporated into polymer matrices. The complex interactions and crosslinking of silica enhance the thermal stability of polymers through molecular dynamics [88].

The thermal stability of BPNCs is primarily contingent upon the composition of the polymer, the structure of the nanocomposites, and the surface interaction characteristics. Additionally, the filler content, fabrication technique, and compatibility of the components can also impact the structure. The alterations in the thermal stability of the polymer matrix resulting from nanofiller modification can be categorized into three principal classifications:

1. A development in thermal stability regarding initial decomposition temperature (T_di_) and maximum degradation value temperature (T_max_).
2. Reducing T_di_ with concurrent diminish of degradation pace.
3. A Degradation of thermal stability concerning T_di_, T_max_, and rate of instability throughout the temperature span [97].

Numerous mechanisms have been postulated to elucidate the manner in which how CNTs enhance the thermal stability of BPNCs. One mechanism is that CNTs delay the weight loss in an inert part by absorbing free radicals or physically adsorbing macromolecules on their surface when the polymer degrades. This decelerates the process of polymer volatilization while maintaining the same rate of decomposition temperature [98, 99]. Another mechanism entails the augmentation of interfacial interaction between the nano-additives and the polymer matrix by CNTs, thereby elevating the activation energy necessary for degradation [100]. One additional mechanism involves the interaction between CNTs and the polymer chains at the interface of the polymer-filler. This interaction leads to an enhancement in the thermal stability of the nanocomposites [101]. The degradation of these particular types of BPNCs is also contingent upon the specific manner in which CNTs have been modified [102]. For instance, CNTs that have been modified with amine groups enhance the thermal stability of epoxy networks through superior reinforcement [103]. This section aims to highlight how the amount and size of the fillers affect the thermal stability of BPNCs.

#### Effect of filler loading on the thermal stability of BPNCs

The inclusion of filler content enhances the effectiveness of PCNs in withstanding high temperatures. Typically, the best thermal stability is achieved when a limited amount of nanoparticles (2–5 wt %) is added, although the specific type of nanoparticles used can affect this. This is because when the nanoparticles are evenly dispersed within the polymer matrix, they create a large surface area of material that does not degrade, thus shielding the polymer chains from thermal breakdown. However, if a higher amount of nanoparticles is added, they tend to clump together, which ultimately lowers the thermal stability of the PCNs [104].

#### Effect of filler size on the thermal stability of BPNCs

Generally, smaller filler particles have a larger surface area, thereby facilitating enhanced dispersion within the biopolymer matrix. The enhanced dispersion can augment the bonding between the filler and the polymer, resulting in a strengthened network and improved thermal stability of the nanocomposite. Smaller particles possess a greater likelihood of generating a a uniform dispersion throughout the matrix, consequently leading to an enhanced thermal stability [105]. Furthermore, diminutive filler particles possess the capability to function as proficient nucleating agents, thereby facilitating crystallization and augmenting the thermal stability of the nanocomposite. Additionally, they can serve as impediments, impeding the diffusion of gases or vapors and increasing the thermal stability of the material [48]. However, beyond a critical filler size range which the thermal stability of the nanocomposites may decrease. This is because excessive filler loading or very small particles can lead to agglomeration or clustering, which may create local stress concentrations. The presence of stress concentrations within the nanocomposite may result in the creation of voids or defects, thereby diminishing its thermal stability. Additionally, the thermal stability of the nanocomposite can be influenced by the specific attributes of the filler, including its surface chemistry and morphology. Consequently, when aiming to attain the desired performance in biopolymer nanocomposites, it is imperative to take into account the combined impact of filler size and other factors on thermal stability [104]. To improve heat resistance, nanoparticles possessing a large surface area capable of attracting radicals and high-polar groups are an appropriate choice. The process involves the nanoparticles attracting and chemically bonding with the volatile degradation substances on their surface, resulting in enhanced thermal stability [106].

### Thermal analysis

Thermal analysis comprises a set of methods that investigate the changes in the characteristics of materials due to heating. The sample is usually in the solid state, and the changes include decomposition, phase transition, melting, and sublimation [105]. Thermal analysis methods include various techniques, The aforementioned techniques encompass Thermogravimetric Analysis (TGA), Differential Thermal Analysis (DTA), Differential Scanning Calorimetry (DSC), Pressurized TGA (PTGA), Thermomechanical Analysis (TMA), Dilatometry (DIL), and Evolved Gas Analysis (EGA). These techniques exhibit variations in terms of the properties they assess. Occasionally, certain methods are merged, such as TGA-DTA. The subsequent methods to be examined are DSC, employed for the detection of the glass transition temperature and melting temperatures, and TGA/DTA, utilized for the evaluation of thermal stability [105].

#### Thermal stability analysis using TGA

TGA is a technique utilized to assess the thermal stability, decomposition temperature, and degradation temperature of a sample by monitoring alterations in its mass upon heating [22, 41, 57, 64, 107, 108]. The TGA curves are plotted with the weight differences of the material along the vertical axis versus time or temperature on the X-axis. As long as a substance is stable, no significant changes in the mass occur. Moreover, TGA determines the critical temperature for a material, beyond which the sample starts to degrade. In polymer studies, since the substance usually melts before decomposing, this technique is employed for the evaluation of the thermal stability of polymers. For instance, TGA can be utilized to assess the stability of a polymer electrolyte, as thermal stability is imperative for ensuring dependable performance and operational safety [107].

**Fig. 7:**
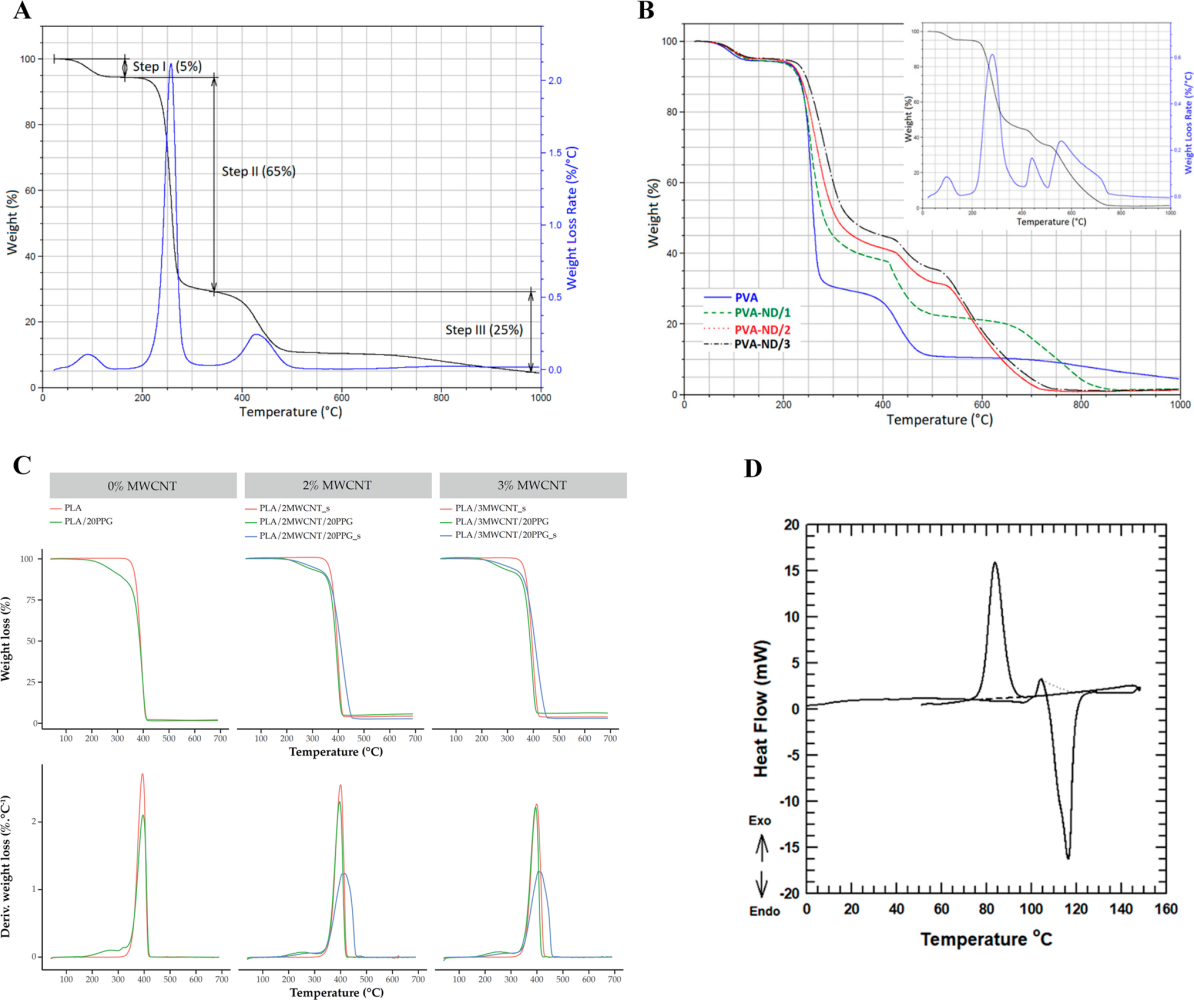
**A** TGA thermograms of the pure PVA [108]. Copyright Reproduced with permission under the Creative Commons Attribution License. Copyright: © 2021 by the authors. Licensee MDPI, Basel, Switzerland. This article is an open access article distributed under the terms and conditions of the Creative Commons Attribution (CC BY) license (https://creativecommons.org/licenses/by/4.0/). **B** Comparison of TGA thermograms for both pristine PVA film and PVA-ND nanocomposites, with an additional focus on the DTGA curve of PVA-ND/3 nanocomposite (including the original TGA curve) [108]. Copyright Reproduced with permission under the Creative Commons Attribution License. Copyright: © 2021 by the authors. Licensee MDPI, Basel, Switzerland. This article is an open access article distributed under the terms and conditions of the Creative Commons Attribution (CC BY) license (https://creativecommons.org/licenses/by/4.0/). **C** TGA thermograms of the pure PLA and composites [110]. Copyright: © 2021 by the authors. Licensee MDPI, Basel, Switzerland. This article is an open-access article distributed under the terms and conditions of the Creative Commons Attribution (CC BY) license (https:// creativecommons.org/licenses/by/ 4.0/). **D** DSC curves of the pure PBS [111]. Copyright: © 2019 by the authors. Licensee MDPI, Basel, Switzerland. This article is an open access article distributed under the terms and conditions of the Creative Commons Attribution (CC BY) license (http://creativecommons.org/licenses/by/4.0/)

Figure 6D illustrates the degradation analysis of a BPNC hydrogel, using TGA, which consists of sodium alginate (SA) as the polymer matrix and graphene oxide (GO) nanosheets crosslinked with zinc [109]. According to the research conducted by Petr [108], it has been determined that the incorporation of NDs into PVA can augment the stability of BPNC. The TGA curves of the PVA sample exhibit three noticeable degradation stages. During the initial phase (Step I), a reduction in weight is noted as a result of the evaporation of water from the film. In the second stage (Step II), weight loss occurs due to PVA dehydration. The third stage (Step III) causes weight loss due to the production of low-molecular-weight compounds (Fig. 7A). The thermal degradation rate of polyvinyl alcohol (PVA) is minimally affected by the presence of non-detectable (ND) particles, as indicated by the TGA analysis presented in Fig. 7B. The films including the pure PVA film, exhibit stability and functionality up to approximately 230 °C, beyond which degradation commences.

To make ductile PLA materials, they were modified with 2-methacryloyloxyethyl isocyanate (MOI). The utilization of a novel sol–gel technique was employed to fabricate polylactic acid (PLA) nanocomposites, resulting in enhanced mechanical properties and heightened thermal stability. TGA analyses were conducted to ascertain the temperature at which thermal decomposition occurs. The thermal decomposition temperature of polylactic acid (PLA) with 5% maleic anhydride oligomer (MOI) and 5–10% silica experiences an increase of 21–32 °C in comparison to pure PLA upon achieving a 10% reduction in weight. This increase can be ascribed to the creation of polymer networks and the presence of inorganic compounds [112]. The PLA/multiwall carbon nanotube (MWCNT) composite filament and PPG, utilized as a plasticizer, were subjected to thermal characterization through TGA analysis. The outcome of this analysis is visually represented in Fig. 7C [110]. In another study conductred by Mofokeng, the ability of PLA/PHBV blends to withstand high temperatures improved by distributing nanoparticles evenly. The TGA results showed that PHBV breaks down at 280 °C and PLA at 364 °C. The thermal stability of PLA/poly (hydroxybutyrate-co-valerate) (PHBV) increased with higher amounts of PLA and the addition of nanoparticles. However, PLA and TiO2 absorbed heat energy causing PHBV to break down at higher temperatures. Evenly dispersed nanoparticles in PLA significantly increased its degradation temperature compared to PHBV [113]. Montmorillonite clay minerals can affect the thermal stability and conductivity of materials. In this study, BPNCs were created using Poly (hydroxybutyrate) (PHB), a biodegradable polymer, and commercially available montmorillonites. Thermal decomposition showed a mass loss of 8% and 4.9% for Na-M at 130 °C and 674 °C, respectively. The percentage of free water loss for 30B-M was determined to be 2% due to two hydroxyl groups in the alkyl ammonium ion. From the thermal stability analysis, it was concluded that only free water influenced nanocomposites during processing [114]. To effectively construct biodegradable NCs, CNT was integrated into a soluble PLA/PEO blend. According to thermogravimetric measurements, the presence of carbon nanotubes (CNT) in the blend significantly augmented its thermal stability in the air environment [115]. The research utilized a conventional melt-mixing techniques to prepare PLA/PEO blend and CNT added NCs. The TGA-scanned graphs revealed that the addition of CNT to the PLA/PEO blend significantly enhanced its thermal stability, resulting in elevated decomposition temperatures [116]. The inclusion of CNT led to a shift in the degradation curves towards elevated temperatures, and an increased concentration of CNT resulted in higher temperatures of degradation. The exceptional thermal stability exhibited by the composites can be ascribed to the heightened thermal stability and potential ability of CNT to scavenge free radicals [115].

#### The differential scanning calorimetry (DSC)

**Fig. 8:**
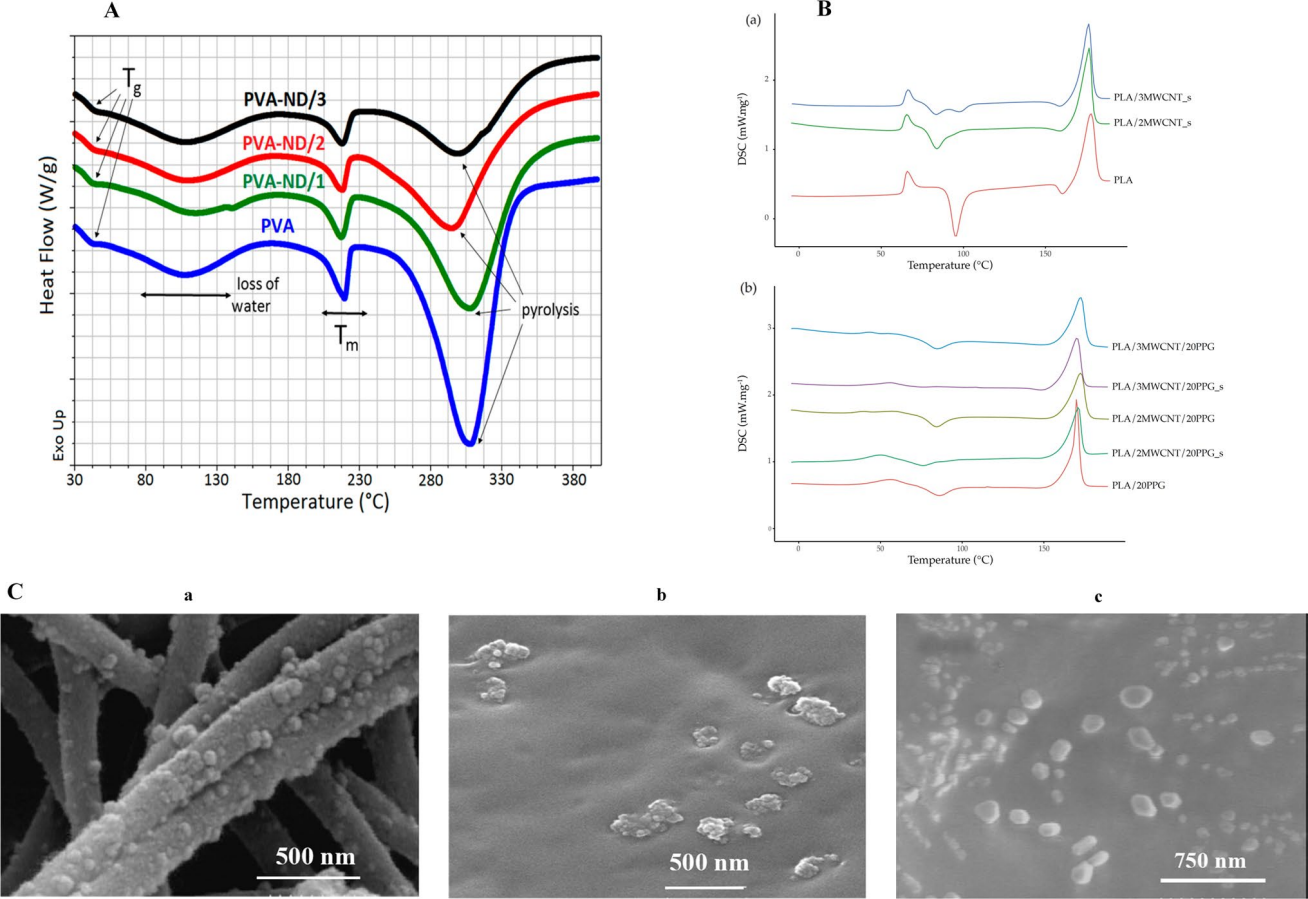
**A** DSC curves of the pure PVA film and the PVA-ND nanocomposites [108]. Copyright Reproduced with permission under the Creative Commons Attribution License. Copyright: © 2021 by the authors. Licensee MDPI, Basel, Switzerland. This article is an open access article distributed under the terms and conditions of the Creative Commons Attribution (CC BY) license (https:// creativecommons.org/licenses/by/4.0/). **B** DSC curve of **A** pure PLA filaments and composite filaments with MWCNT, and **B** composite filaments with PPG as plasticizer [110]. Copyright: © 2021 by the authors. Licensee MDPI, Basel, Switzerland. This article is an open-access article distributed under the terms and conditions of the Creative Commons Attribution (CC BY) license (https:// creativecommons.org/licenses/by/ 4.0/). **C** SEM micrographs illustrating the distinctive morphologies of various BBPNCs developed: (*a*) Surface deposition of Zeolitic imidazolate frameworks (ZIF-8)/chitosan BPNCs as electrospun films on AZ91 magnesium alloy [117]. (*b*) Nanodiamond/PCL-based BPNCs, and (*c*): Nanobioglass/PCL-based BPNCs, both prepared via a three-dimensional printing technique, for their application as tissue engineering scaffolds

DSC can assess the impact on heat due to properties such as dielectric behavior, chemical reactions, and changes in phase throughout temperature variations. This occurs when nanofillers interact with the polymer matrix, which causes the alterations in the polymer matrix's structure. The exothermic transition means that less heat flux is required to raise the temperature of the sample than the reference materials during analysis, and the exothermic peaks are upward. On the other hand, the endothermic transition requires more heat flux with downward peaks. Immiscible materials, such as PC/PS blends, have two Tg, according to previous reports, because these two polymers are immiscible [38]. The DSC graph of pure Poly Butylene Succinate (PBS) is shown in Fig. 7D [111]. According to a study by Khutia et al., adding Al_2_O_3_ to a mixture of polycarbonate (PC) and polystyrene (PS) increased the glass transition temperature (Tg) and melting temperature (Tm). However they decreased the degree of crystallinity as the concentration of Al_2_O_3_ increased. The interaction between Al_2_O_3_ and PC/PS blend system has the potential to reduce crystallinity, as evidenced by shifts in Tg and Tm values on the differential scanning calorimetry (DSC) curve. Another study by B. Petr (as shown in Fig. 8A) found that increasing the loading of nanodiamonds (NDs) in the polyvinyl alcohol (PVA) matrix slightly raised the Tg of the PVA/ND nanocomposite. The rise in glass transition temperature (Tg) can plausibly be ascribed to the partial crosslinking of polyvinyl alcohol (PVA) facilitated by the presence of nanodiamonds (NDs) (Fig. 8A) [108].

The DSC curve in Fig. 8B. indicated that the Tg and melting temperature (TM) of PLA/20PPG were lower compared to pure PLA. The observed disparity can be ascribed to the incorporation of PPG within the composite materials, thereby serving as a plasticizer. The T_M_ and Tg values of pure PLA remained unaffected [110]. One prominent endothermic peak was associated with CS, which absorbed moisture. In contrast, another minor peak at 210 °C was related to PVA melting temperature. All of the BPNCs exhibited comparable DSC curves to those of PVA and CS, albeit slight inconsistencies exists in the endothermic melting transition. These discrepancies can possibly be ascribed to the interactions taking place between the polymers and the nanofillers. As a result, the inclusion of GO and NiO into the polyvinyl alcohol/chitosan/graphene oxide/nickel oxide (PVA/CS/GO/NiO) nanocomposites brought about modifications in their thermal properties [41]. The DSC thermogram displayed three distinct transitions. The initial endothermic transition was observed within a limited temperature range of 22–24 °C. The incorporation of Al_2_O_3_, the transition was shifted to a higher temperature. Between the temperatures of 135 and 136.5 °C, the second transformation took place, which corresponds to the glass transition temperature (Tg). The miscibility of the blend system was confirmed by the presence of a solitary Tg. The inclusion of Al_2_O_3_ was observed to cause a shift in the Tg point to a lower temperature. The alterations in Al_2_O_3_ concentration, Tg, and Tm in the DSC curves were attributed to the interaction between PC, PS, and Al_2_O_3_ [38].

### Recent advances in thermal properties of BPNCs

A recent development in the thermal properties of bio-based polymer nanocomposites (BPNCs) involves improving their ability to degrade at a customized rate in natural environments. The incorporation of nanoparticles into biopolymers is an important factor in the creation of these eco-friendly materials. This is mainly because the nanoparticles have a high aspect ratio, allowing them to have important effects even at low concentrations (≤ 5%) [118]. Additionally, the state of dispersion of nanofillers in the matrix is a paramount concern as it directly influences their surface area available for interaction with the matrix. Notably, when nanodiamonds are adequately dispersed, they have the potential to enhance thermal stability, strength, and toughness [119].

One of the recent advances is the use of nanocellulose, which is derived from cellulose nanofibers or nanocrystals, as a reinforcing material in biopolymer nanocomposites. Nanocellulose exhibits exceptional mechanical and thermal characteristics, and its integration into biopolymer matrices augments the thermal durability and potency of the resultant nanocomposites. Additionally, nanocellulose has the potential to enhance the barrier properties, flame retardancy, and thermal conductivity of biopolymer nanocomposites [120].

Another important recent advance is the utilization of graphene and other carbon-based nanomaterials in biopolymer nanocomposites. Graphene possesses extraordinary thermal conductivity and has the potential to enhance the thermal characteristics of biopolymer matrices. The incorporation of graphene into biopolymer nanocomposites leads to enhanced thermal stability, increased thermal conductivity, and improved flame retardancy [106]. Besides, the incorporation of clay nanoparticles into biopolymer matrices has been examined as a strategy to enhance the thermal characteristics of the resultant nanocomposites. The dispersion of clay nanoparticles within the biopolymer matrix has the potential to augment the thermal stability, flame retardancy, and mechanical strength of the nanocomposites [121, 122].

Functionalized nanoparticles, like metal oxide nanoparticles, carbon nanotubes, or nanofibers, can improve the thermal properties of biopolymer nanocomposites by establishing strong interfacial interactions with the biopolymer matrix, leading to enhanced thermal stability, mechanical strength, and heat resistance [123]. In addition, The thermal transport of the BPNCS polymer matrix is improved through the integration of fillers possessing exceptional thermal conductivity, such as boron nitride, diamond, or carbon-based particles including graphite, carbon nanotubes (CNTs), and graphene (GNPs) [71].

## Biodegradable polymer nanocomposite's fabrication techniques

Various fabrication techniques have been developed for the creation of polymer nanocomposites. These techniques aim to produce nanocomposite materials with uniform dispersion and without aggregation. Some of the most common fabrication techniques include:

### Melt-mixing or blending

This method involves mixing the polymer and nanoparticles in the molten state, ensuring a homogeneous distribution of the nanoparticles throughout the polymer matrix [124]. Various fabrication techniques have been developed for the creation of polymer nanocomposites. These techniques aim to produce nanocomposite materials with uniform dispersion and without aggregation. Some of the most common fabrication techniques include:

### Mixing

This technique involves the mechanical mixing of the polymer and nanoparticles, which can be done using high-shear mixing, ultrasound, or other mixing methods, The mixture is then evaporated to obtain a composite material [124].

### In-situ polymerization

In this method, the nanoparticles are present during the polymerization process, allowing for the formation of a strong bond between the polymer and nanoparticles [124].

### Solution dispersion

This technique involves the dispersion of nanoparticles in a solvent, followed by the addition of the polymer to the solution. This method allows for a more uniform distribution of the nanoparticles in the polymer matrix [125].

### Intercalation

This method is based on the exfoliation of layered silicates, such as montmorillonite and mica, which can be used as nanoparticles. The intercalation process allows for a more uniform distribution of the nanoparticles in the polymer matrix [126].

### Melt-compounding

This simple melt-compounding-based approach avoids the need for surface modification of nanofillers and is compatible with current industrial processes, such as extrusion and injection molding. This method is environmentally benign due to the absence of organic solvents, organic surfactants, and a variety of specific chemical substances required for complicated polymerization and sol–gel processes [126].

### Electrospinning

In this technique, an electric field is used to draw out polymer fibers from a solution or melt. Nanoparticles can be incorporated into the fibers during the spinning process, resulting in a nano-composite structure [49, 127].

Each of these fabrication techniques has its advantages and disadvantages, and the choice of method depends on the specific requirements of the polymer nanocomposite being developed.

## Biodegradable polymer nanocomposite's characterization techniques

Once the polymer nanocomposites are fabricated, various characterization techniques are employed to assess their properties. These characterizations help in understanding the structure, morphology, and performance of the nanocomposites. Some commonly used characterization techniques include:

### Morphological analysis

Morphological analysis techniques such as scanning electron microscopy (SEM) and transmission electron microscopy (TEM) allow for the observation and characterization of the nanocomposite's microstructure, including the dispersion and distribution of nanoparticles within the polymer matrix. TEM Provides high-resolution images, allowing visualization of the nanostructure and confirming the dispersion of nanofillers. SEM Offers detailed surface morphology information, aiding in the observation of the composite's overall structure [128, 129].

### Mechanical testing

Mechanical testing methods such as tensile testing, flexural testing, and impact testing can evaluate the mechanical properties of the nanocomposite, including its strength, stiffness, toughness, and resilience [128]**.**

### Fourier transform infrared spectroscopy (FTIR)

Analyzes chemical bonds and functional groups, helping to confirm the presence of nanofillers and assess chemical interactions. While X-ray Diffraction (XRD) analysis can identify and quantify the crystalline phases present in the nanocomposite, providing information about the degree of crystallinity and the crystal structure of the material. Rheological testing examines the flow and deformation properties of the nanocomposite, providing insights into its processability and rheological behavior [125, 128].

## Application of biodegradable polymer nanocomposites

Electronic technology is developing rapidly and changing our lives. A wide variety of electronics has been widely used in every field, such as telecommunications, manufacturing, entertainment, healthcare, etc. However, increased electronic waste (E-waste) has also caused a serious global environmental pollution problem. The growing apprehension towards environmental issues has prompted the investigation of alternative materials as a substitute for traditional materials, not only for E-waste management but also to facilitate the biomedical applications of transient electronic devices [130]. Surface modification of orthopedic implants by bioactive BPNC film [127], tissue engineering, and drug-eluting composite scaffolds based on BPNCs are some of the recent developments by Tamjid et al. [49, 131, 132]. SEM morphology images of some of these scaffolds are presented in Fig. 8C.a–c.

Biodegradable polymers constitute a category of materials that possess numerous commendable attributes, including their lightweight nature and ease of fabrication, among others [122]. However, a fundamental problem with using this material is the lack of thermal and electrical conductivity in their properties. A practical method to address this problem is to manufacture BPNCs through the incorporation of conductive particles into an insulating polymer matrix. Guo et al. have made advancements in enhancing the thermal and electrical conductivity by introducing graphene nano-plates (GNPs) into poly (butylene adipate-co-butylene terephthalate) (PBAT). In order to establish a connected network, the dispersion of GNPs within the PBAT matrix was carefully regulated through blending with PLA. By incorporating 40 wt% of GNPs, composites exhibiting an electrical conductivity of 338 S/m and a thermal conductivity of 3.15 W/m⋅K were successfully fabricated. This BPNC exhibits promising potential for application within the electronic industry [88].

**Fig. 9:**
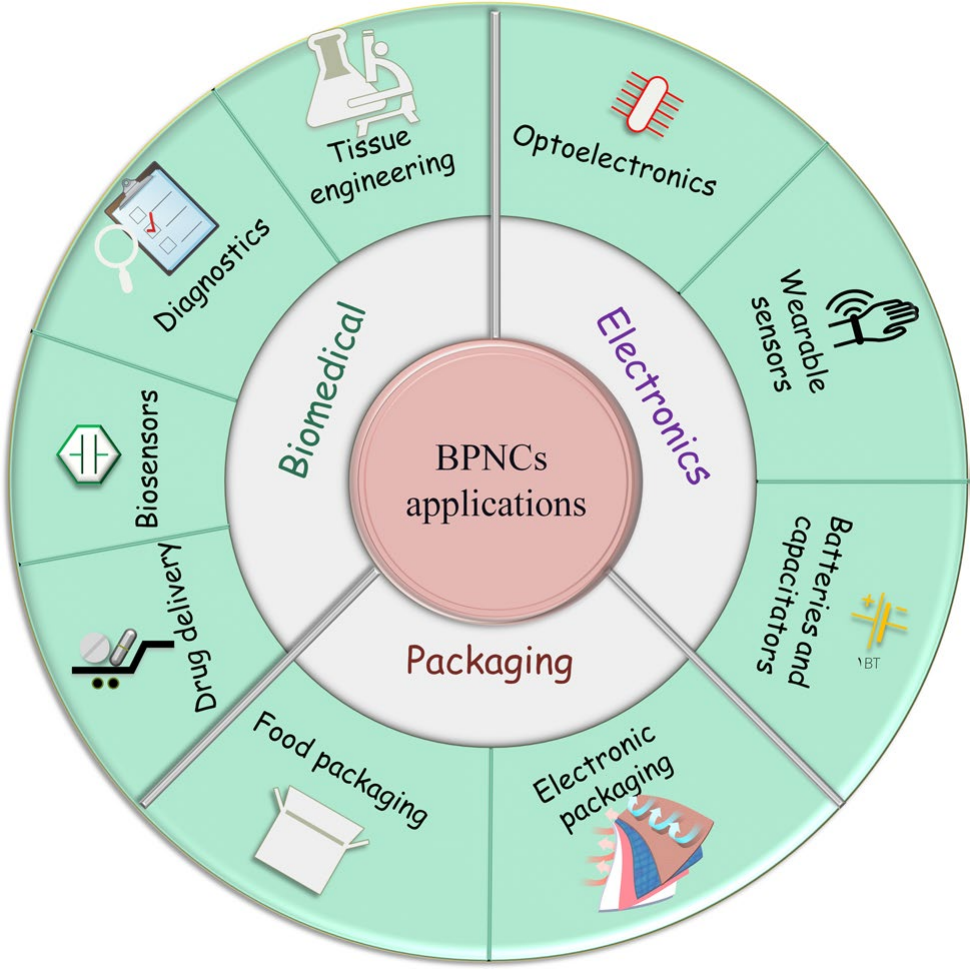
Commercial applications of BPNCs in different fields

Biodegradable electronic devices have been employed across diverse sectors, including biomedical applications and tissue engineering [133], and electronic packaging [134]. In addition, they have been used in diagnostic platforms [135], therapeutic devices applications, and power supply [136], in biomedical applications. In diagnostic applications, transient electronics are very efficient due to their close junction with target areas in tissue and organs and can find abnormal signals in early states [137] (Fig. 9). Boutry et al. [135] successfully developed pressure and strain sensors utilizing biodegradable materials. These sensors possess the capability to accurately measure both strain and pressure, employing two vertically separated sensors that operate independently without any interference. In this structure, poly (octa methylene maleate (anhydride) citrate)) (POMaC) is a soft, stretchable, biodegradable, elastomeric biomaterial used for the strain sensor and packaging. Two comb-like thin-film electrodes slide relative to each other to measure the capacitance change and determine the strain. For pressure sensing, the capacitance variation depends on the distance between the upper and lower electrodes. The dielectric layer made of biodegradable elastomer poly (glycerol sebacate) (PGS) enhances the high-pressure sensor sensitivity and produces a fast response time. This sensor has shown excellent biocompatibility and function in an in vivo study and the potential applicability to the real-time monitoring of tendon healing [135].

Biodegradablee electronic conducting polymeric scaffolds are especially attractive for nerve tissue regeneration. In the study of Shafei et al. [133], electrospun PCL nanofibers with 92% porosity coated by polypyrrole (PPy) were fabricated. The PCL fibers underwent a transformation resulting in an increase in electrical conductivity from 1.3 to 1.9 S/cm. Additionally, they exhibited excellent biocompatibility, facilitating the growth of neural-like cells. Furthermore, the coated PCL scaffold demonstrated superior cell affinity when compared to the non-coated counterpart [135]. Generally; electronic devices are comprised of a substrate and a variety of functional components, including semiconducting layers, dielectric layers, electrodes, and capsulations. The main purpose of the substrate in electronic devices is to provide support for the other layers that possess different electrical characteristics. To avoid unwanted interference, the substrate must effectively isolate the electronic elements. Polymers are commonly employed in applications that necessitate insulation properties, like substrates, as they possess a high ability to endure electrical stress without breaking down.

Biodegradable polymers, such as melanin, PANI, PPy, and PPDOT, can be used as conducting or semiconducting layers. However, these conductive and even insulating polymers can become more conductive by adding conductive nanofillers like graphene and CNTs [16]. In the dielectric part, a material with electrically insulating properties can be used. When subjected to an electric field, these materials exhibit a phenomenon wherein electric charges, which do not traverse the dielectric material, undergo a slight displacement from their average equilibrium positions. This limited charge mobility results in the creation of an internal electric field that opposes the direction of the externally applied electric field, thereby diminishing the overall electric field within the dielectric material. Dielectrics find application in capacitors, making them indispensable for capacitive sensing and field-effect transistors [138].

### Electronic packaging applications

Biodegradable polymers, as packaging materials, have low barrier properties. To overcome this problem, it is necessary to use additive nanoparticles to enhance mechanical/thermal resistance, oxidation stability, biodegradability, reduced gas permeability, and flammability [139]. In electronic devices, the packaging is essential to prevent gas and water vapor because (semi) conductors have organic compounds that easily oxidize and lose their activity. Biodegradable polymers are of interest in providing this barrier property due to their high hydrophobicity and crystallinity [140]. A study by Chen et al. fabricated core–shell nanostructure AgNWs@SiO_2_ and used it as 1D thermal conductive fillers, which improved the thermal conductivity of the epoxy matrix. Silver nanowires (AgNWs) possess high thermal conductivity, but do not maintain homogeneous distribution in an epoxy matrix and due to their limited electrical conductivity they are not suitable for electronic packaging. To overcome the incompatibility between AgNWs and the epoxy, a silica nanolayer was applied on AgNWs. The thermal conductivity of epoxy/AgNWs@SiO_2_ composite increased from 0.19 to 1.03 W/mK. As the silica nanolayer led to electrical insulation of the composite, this composition is potentially of interest in electronic packaging [134].

Energy storage devices can be classified into batteries and supercapacitors, which include electrodes, electrolyte, and separators. The electrolyte has a substantial role in electrochemical characteristics. Supercapacitors, with the ability to charge and discharge rapidly and have high power density, have attracted much attention. To achieve better ionic conductivity and suitable power density, as well as flexibility, BPNCs are good compositions to replace traditional of [141]. The dielectric constant value of PC and PS is 3 and 2.6, respectively. In another study, excellent optical and mechanical properties were achieved by PC/PS blend [142]. Aluminum oxide (Al_2_O_3_) was added to polycarbonate (PC) and polystyrene (PS) to create the optimal dielectric medium, due to its high thermal conductivity and dielectric constant value (ε = 9.4). It was observed that the dielectric parameters were influenced by external direct current potential, frequency, and alumina loading. Notably, the composite containing 15% Al_2_O_3_ exhibited a dielectric constant value of 3.5 × 105 at 10 Hz, rendering it suitable for various engineering applications.

### Optoelectronic applications

Cadmium oxide (CdO) is classified as a transparent conducting oxide (TCO) and possesses semiconductor characteristics. CdO nanoparticles (NPs) exhibit great potential as viable options for applications in photovoltaic cells, photo-transistors, photo-diodes, and gas sensors. It is worth noting that the chemical properties of CdO are influenced by the synthesis process. Consequently, the reduction in particle size can bring about alterations in the electronic and optical properties. In the research by Lokanatha Reddy et al., they used PEG as a surface modification and passivation agent for CdO NPs. This nanocomposite can be useful in optoelectronic applications, as it reduces the non-radiative recombination centers and enhances the fluorescence intensity [143].

## Future prospective

Environmental pollution caused by oil-based polymeric materials poses a substantial threat to the ecosystem. The remarkable potential of nanocomposites can be exemplified by the substantial investments made by numerous companies and administrations across the globe. As a result, nanocomposites are expected to exert a considerable influence on the global economy and trade. These nanocomposites are the next generation of the materials market. PL composites have emerged as the most promising material after reinforcement, but reducing thermal degradation during processing is necessary. The requirement to improve some properties and reduce costs is still of interest in some of these nanocomposites.

CNT-based nanocomposites will replace current conductive materials already being used in the field of electrical and electronics industries. These materials include BPNCs that are technologically advanced as versatile materials, especially for flexible electronic components and nanocomposite devices designed and manufactured for use in solar panels, energy storage capacitors, and UV protectors. Nanocomposites of PS/RGO exhibiting advanced dielectric properties, synthesized through a cost-effective and uncomplicated process, hold potential for deployment in a wide range of flexible and biocompatible electronic components. The electronics industry has been growing over the last decades. The use of various electronic devices is increasing day by day. These electrical devices need higher frequencies for good performance, and thus their performance is disrupted by electromagnetic interference signals. The use of products and devices that emit high-frequency electromagnetic radiation is increasing. These waves are harmful to human health. Previously, protective metals that worked well were used. To improve protective materials, researchers are developing novel nanocomposites with better dielectric strength, process flexibility, and low fabrication temperatures. These new materials are expected to provide superior protection against environmental hazards compared to conventional materials.

Bioplastics have been developed as a potential solution to reduce the negative impact caused by traditional plastics. According to the European Bioplastics Organization, a plastic material can be classified as a bioplastic if it has either biobased, biodegradable, or both properties [17, 144]. The main limitation for using BPNCs is that they are 20–80% more expensive than conventional plastics. Solvent casting is usually more popular for making BPNCs. The synthesis of biodegradable plastics is costly, herefore, they are not as commercialized as petroleum-based plastics, which has limited the market penetration of BPNCs. Two factors are essential to improve dielectric, electrical, thermal, and physical properties: the distribution of nanofillers and the adhesive strength of the interface. If the manufacturing costs can be reduced while improving the properties, they can undoubtedly perform well in the global market. minimizing the thermal degradation of the initial polymer matrix during the processing stage is vital. Various methods are used to modify materials. Among them, the most useful ones include cross-linking, electrospinning, blending, sol–gel processing, and grafting, each of which has its limitations.

The study of BPNCs is a captivating field of interdisciplinary research that encompasses the disciplines of physics, chemistry, biology, materials science, and engineering. The amalgamation of knowledge from scientists with diverse backgrounds is poised to yield novel scientific discoveries, particularly in the realm of materials science, where macroscopically engineered materials can be created through nanostructures. It is anticipated that the global market for biodegradable plastics will experience a substantial growth from $7.7 billion in 2021 to $23.3 billion in 2026. It has an array of favorable applications, though the most prominent are the packaging, coating, automotive, and electronics sectors that are at the forefront of using these new materials. BPNCs can be programmed and manufactured based on specific requirements. In this way, a particular conductive nanoparticle is usually combined with metal oxide in a polymer matrix, and several fillers are provided in the polymer matrix to meet a specific requirement. Other promising areas for Future research are low-cost amplifiers, cheap polymeric materials, and abundant availability.

BPNCs seem to be advantageous in diverse contexts, including the electronics and chemical industries, the aerospace and transportation industries, medicine, healthcare, and the environment. Consequently, it is anticipated that nanocomposites will enhance the overall quality of human existence in the foreseeable Future. Given the increasing need for robust industrial systems, such as nanofillers and their composites, and the various cost-effective sectors they cater to, optimistic projections appear attainable. A large number of these products are consumer goods and are therefore expected to stabilize their projected market. For example, electrically conductive polymers, nano-smart switches, and automotive sensors are currently used by General Electrics and Cabot in the United States since 2002. Meanwhile, General Motors has effectively employed nanocomposites comprising clay and talc in their structural components. BASF (Germany), NatureWorks (USA), Total Corbion (Netherlands), Novamont (Italy), Biome Bioplastics (UK), Mitsubishi Chemical Holding Corporation (Japan), and Toray Industries (Japan), have also utilized similar materials in their respective operations. Plantic Technologies (based in Australia), Danimer Scientific (located in the US), and Fkur Kunstsoff (a German company) are among the notable participants in the global market for biodegradable plastics.oo.

## Conclusions

The demand for recyclable materials possessing distinct characteristics, including thermal, electrical, and dielectric properties, has stimulated the emergence of novel functional materials for eco-friendly technologies. Biodegradable polymer nanocomposites (BPNCs), often regarded as the Future materials, have recently gained important attention in fabricating eco-friendly materials with desired properties for versatile applications. The main industrial sectors of interest are medical applications, sensors, catalysis, and energy conversion and storage (i.e., batteries, supercapacitors, dielectric capacitors, solar cells), biomedical devices, tissue engineering, anti-static agents, optical and electroactive devices.

Both natural polymers, such as chitosan and cellulose, and synthetic polymers, including PLA, PVA, PVP, and PCL, have been utilized as matrices. The incorporation of filler materials possessing nanometric dimensions and engineered morphology into the polymer matrix is crucial for the customization of properties. Many nanomaterials, including metallic and inorganic nanoparticles, and carbon nanostructures, have been used. The electrical and thermal properties of BPNCs are significantly influenced by the composition, size, morphology of the nanofillers, and the quantity of additives. Moreover, the thermal, physical, dielectric, and electrical properties of BPNCs are significantly influenced by the distribution of nanofillers and the strength of the interface adhesion. The amount of nanofillers should be finely controlled to achieve improvement and optimization of BPNCs properties. For instance, if the mechanical strength is of concern, in order to prevent the accumulation of particulate materials and the formation of a network, it is imperative that the concentration of fillers remains below the percolation threshold.. In contrast, if the electrical conductivity or thermal conductivity is required, the formation of conductive networks would be beneficial for targeted properties.

The dielectric properties of a polymer matrix are generally affected by various factors, including the atomic, electronic, interfacial, and orientation polarization of the matrix molecules. Additionally, the dielectric chemical structure and polarization within the BPNC are influenced by the interfacial interaction between the nanofiller and the polymer. An Alpha dielectric analyzer is a convenient high-resolution technique that is used to analyze the charge transport and measure the dielectric properties of BPNC films in broad frequency and temperature ranges. Other techniques include dielectric relaxation spectroscopy (DRS), broadband dielectric spectroscopy (BDS), impedance analyzer, and rectangular waveguide adapter technique.

Atomic defects, random orientation, impurities, vacancies, voids, and entanglement of polymer chains are all factors that impede the thermal conductivity of polymers. Overall, a higher number of conductive fillers results in higher final thermal conductivity. Moreover, the fillers dispersion and geometry are other primary considerations. Additionally, the thermal stability of BPMCs is of paramount importance in determining their overall performance. Thermal analysis methods for thermal properties consist of TGA, DTA, DSC, PTGA, TMA, DIL, and EGA.

As a part of the implications of this emerging technology, other aspects of BPNCs need to be carefully considered when dealing with nanoparticles. It is imperative to acknowledge that the release of nanoparticles into the environment poses a substantial health and safety concern. Consequently, there exists a pressing requirement for research on the release of nanofillers, nanoparticles, and their associated adverse effects to guarantee the sustainable and secure implementation of BPNCs in the Future.

## Data Availability

There are no data available.

## Code availability

There are no code available.

## References

[1] Xu P, Liu X, Zhao Y, Lan D, Shin I (2023). Study of graphdiyne biomimetic nanomaterials as fluorescent sensors of ciprofloxacin hydrochloride in water environment. Desalin Water Treat.

[2] Scaffolds B, Xu Y, Zhang F, Zhai W, Cheng S, Li J, Wang Y, Unraveling of advances in 3D-printed polymer-based; 2022 10.3390/polym14030566 PMC8840342 35160556

[3] Polymers for Advanced Techs - 2019 - Shojaie - Electrospun electroactive nanofibers of gelatin‐oligoaniline Poly vinyl.pdf, (n.d.)

[4] Zhang S (2022). Auger scattering dynamic of photo-excited hot carriers in nano-graphite film. Appl Phys Lett.

[5] Rostamian M, Hosseini H, Fakhri V, Yousefi P, Farahani M, Jalali A, Goodarzi V, Su C (2022). Chemosphere Introducing a bio sorbent for removal of methylene blue dye based on flexible poly (glycerol sebacate)/chitosan/graphene oxide ecofriendly nanocomposites. Chemosphere.

[6] Preethikaharshini J, Naresh K, Rajeshkumar G, Arumugaprabu V, Khan MA (2022). Review of advanced techniques for manufacturing biocomposites: non-destructive evaluation and artificial intelligence-assisted modeling. J Mater Sci.

[7] Bari SS, Chatterjee A, Mishra S (2016). Biodegradable polymer nanocomposites: an overview. Polym Rev.

[8] Mclauchlin AR, Thomas NL (2012). Biodegradable polymer nanocomposites. Adv Polym Nanocomp Types Appl.

[9] Reshmy R, Philip E, Vaisakh PH, Sindhu R, Binod P, Madhavan A, Pandey A, Sirohi R, Tarafdar A (2021). Biodegradable polymer composites. Biomass, Biofuels, Biochem.

[10] Yang S, Zhang Y, Sha Z, Huang Z, Wang H, Wang F (2022). Deterministic manipulation of heat flow via three-dimensional- printed thermal meta-materials for multiple protection of critical components. ACS Appl Mater Interfaces.

[11] Wang H, Huang Z, Zeng X, Li J, Zhang Y, Hu Q (2023). Enhanced anticarbonization and electrical performance of epoxy resin via densified spherical boron nitride networks. ACS Appl Electr Mater.

[12] Yang Y, Zhang Z, Zhou Y, Wang C, Zhu H (2023). Design of a simultaneous information and power transfer system based on a modulating feature of magnetron. IEEE Trans Microw Theory Tech.

[13] Yang B, Wang H, Zhang M, Jia F, Liu Y, Lu Z (2023). Mechanically strong, flexible, and flame-retardant Ti_3_C_2_T_x_ MXene-coated aramid paper with superior electromagnetic interference shielding and electrical heating performance. Chem Eng J.

[14] Du S, Xie H, Yin J, Fang T, Zhang S, Sun Y, Cai C, Bi G, Chen Z, Xiao D, Chen W, Yang X, Wang D, Yin W (2023). Competition pathways of energy relaxation of hot electrons through coupling with optical, Surface, and Acoustic Phonons. J Phys Chem C..

[15] Huang Y, Kormakov S, He X, Gao X, Zheng X, Liu Y, Sun J, Wu D (2019). Conductive polymer composites from renewable resources: an overview of preparation, properties, and applications. Polymers (Basel).

[16] Liu H, Jian R, Chen H, Tian X, Sun C, Zhu J, Yang Z, Sun J, Wang C (2019). Application of biodegradable and biocompatible nanocomposites in electronics: current status and future directions. Nanomaterials.

[17] Mahmud S, Hasan KMF, Jahid MA, Mohiuddin K, Zhang R, Zhu J (2021). Comprehensive review on plant fiber-reinforced polymeric biocomposites. J Mater Sci.

[18] Idumah CI, Hassan A, Moniruzzaman M, Winey KI, Lee JY, Liao Y, Nagahata R, Horiuchi S, Guo Y, Zuo X, Xue Y, Tang J, Gouzman M, Fang Y, Zhou Y, Wang L, Yu Y, Rafailovich MH, Rancourt JD, Taylor LT, Pandey P, Mohanty S, Nayak SK, Bershtein VA, Egorova LM, Yakushev PN, Pissis P, Sysel P, Brozova L, Yang J, Lin Y, Wang JJ, Lai M, Li J, Liu J, Tong X, Cheng H, Mofokeng JP, Luyt AS, Araújo A, Botelho G, Oliveira M, Machado AV, Chatterjee A, Deopura BL, Marosföi BB, Marosi GJ, Szép A, Anna P, Keszei S, Nagy BJ, Martvonova H, Sajó IE, Chen XX, Wang JJ, Lin M, Zhong W, Feng T, Chen XX, Chen J, Xue F, Nilagiri Balasubramanian KB, Ramesh T, Chen BK, Shih CC, Chen AF, Bikiaris D, Leung SN, Sawada T, Ando S, Botana A, Mollo M, Eisenberg P, Torres Sanchez RM, Burger N, Laachachi A, Ferriol M, Lutz M, Toniazzo V, Ruch D, Chipara MM, Lozano K, Hernandez A, Chipara MM, Di Blasi C, Galgano A, Branca C (2006). Engineering thermally and electrically conductive biodegradable polymer nanocomposites. Compos Part B Eng.

[19] Hosseini H, Mohammad S, Wurm FR, Goodarzi V (2021). Display of hidden properties of flexible aerogel based on bacterial cellulose/polyaniline nanocomposites with helping of multiscale modeling. Eur Polym J.

[20] Dhayal V, Hashmi SZ, Kumar U, Choudhary BL, Kuznetsov AE, Dalela S, Kumar S, Kaya S, Dolia SN, Alvi PA (2020). Spectroscopic studies, molecular structure optimization and investigation of structural and electrical properties of novel and biodegradable Chitosan-GO polymer nanocomposites. J Mater Sci.

[21] Ciuprina F, Andrei L, Stoian S, Gabor R, Panaitescu D, Dielectric Response and Dynamic Mechanical Analysis of PHBV-TiO_2_ Nanocomposites, In: Proc. 2020 IEEE 3rd Int. Conf. Dielectr. ICD 2020. (2020) 201–204. 10.1109/ICD46958.2020.9341988.

[22] Deshmukh K, Ahamed MB, Deshmukh RR, Pasha SKK, Sadasivuni KK, Polu AR, Ponnamma D, AlMaadeed MAA, Chidambaram K (2017). Newly developed biodegradable polymer nanocomposites of cellulose acetate and Al_2_O_3_ nanoparticles with enhanced dielectric performance for embedded passive applications. J Mater Sci Mater Electron.

[23] Deshmukh K, Ahamed MB, Sadasivuni KK, Ponnamma D, AlMaadeed MAA, Deshmukh RR, Pasha SKK, Polu AR, Chidambaram K (2017). Fumed SiO_2_ nanoparticle reinforced biopolymer blend nanocomposites with high dielectric constant and low dielectric loss for flexible organic electronics. J Appl Polym Sci.

[24] Abou Hammad AB, Abd El-Aziz ME, Hasanin MS, Kamel S (2019). A novel electromagnetic biodegradable nanocomposite based on cellulose, polyaniline, and cobalt ferrite nanoparticles. Carbohydr Polym.

[25] Muthupandeeswari A, Kalyani P, Vickraman P (2020). Evaluation of vital features of PVA–CaCO_3_ nanocomposite films for biodegradable packaging applications. Polym Bull.

[26] Patel GB, Singh NL, Singh F, Kulriya PK (2021). Effect of swift heavy ions irradiation on physicochemical and dielectric properties of chitosan and chitosan-Ag nanocomposites. Radiat Phys Chem.

[27] Choudhary S (2018). Characterization of amorphous silica nanofiller effect on the structural, morphological, optical, thermal, dielectric and electrical properties of PVA–PVP blend based polymer nanocomposites for their flexible nanodielectric applications. J Mater Sci Mater Electron.

[28] Fan Y, Huang X, Wang G, Jiang P (2015). Core-shell structured biopolymer@BaTiO_3_ nanoparticles for biopolymer nanocomposites with significantly enhanced dielectric properties and energy storage capability. J Phys Chem C.

[29] Youssef AM, El-Aziz MEA, Abd El-Sayed ES, Moussa MA, Turky G, Kamel S (2019). Rational design and electrical study of conducting bionanocomposites hydrogel based on chitosan and silver nanoparticles. Int J Biol Macromol.

[30] Mohammed J, Abubakar BF, Yerima KU, Hamisu H, Ismail UT, Muhammad A, Zulfatu UF, Abubakar A, Salihu NM, Abubakar MS, Saidu Y, Tchouank Tekou Carol T, Srivastava AK (2018). Biodegradable polymer modified rGO/PANI/CCTO nanocomposites: structural and dielectric properties. Mater Today Proc.

[31] Chen H, Li X, Yu W, Wang J, Shi Z, Xiong C, Yang Q (2020). Chitin/MoS_2_ nanosheet dielectric composite films with significantly enhanced discharge energy density and efficiency. Biomacromol.

[32] Ahmad AF, Aziz SA, Obaiys SJ, Zaid MHM, Matori KA, Samikannu K (2020). U.S as Aliyu, Biodegradable poly (lactic acid)/poly (ethylene glycol) reinforced multi-walled carbon nanotube nanocomposite fabrication, characterization, properties, and applications. Polymers (Basel)..

[33] Papageorgiou GZ, Terzopoulou Z, Bikiaris D, Triantafyllidis KS, Diamanti E, Gournis D, Klonos P, Giannoulidis E, Pissis P (2014). Evaluation of the formed interface in biodegradable poly(l-lactic acid)/graphene oxide nanocomposites and the effect of nanofillers on mechanical and thermal properties. Thermochim Acta.

[34] Dhatarwal P, Sengwa RJ, Choudhary S (2020). Multifunctional (PVP/PEO)/SnO_2_ nanocomposites of tunable optical and dielectric properties. Optik (Stuttg).

[35] Aziz SB, Brza MA, Mohamed PA, Kadir MFZ, Hamsan MH, Abdulwahid RT, Woo HJ (2019). Increase of metallic silver nanoparticles in Chitosan:AgNt based polymer electrolytes incorporated with alumina filler. Results Phys.

[36] Fryn P, Lalik S, Górska N, Iwan A, Marzec M (2021). Comparison of the dielectric properties of ecoflex® with l, d-poly(Lactic acid) or polycaprolactone in the presence of swcn or 5cb. Materials (Basel).

[37] Guzmán Sierra DL, Bdikin I, Tkach A, Vilarinho PM, Nunes C, Ferreira P (2021). Flexible piezoelectric chitosan and barium titanate biocomposite films for sensor applications. Eur J Inorg Chem.

[38] Khutia M, Joshi GM, Deshmukh K, Pandey M (2015). Optimization of dielectric constant of polycarbonate/polystyrene modified blend by ceramic metal oxide. Polym - Plast Technol Eng.

[39] Hashim A, Hadi A (2018). Made from nanocomposites ( biodegradable polymers – metal oxide nanoparticles ). Ukrainian J Phys.

[40] Muzaffar A, Ahamed MB, Deshmukh K, Pasha SKK (2020). Dielectric properties and electromagnetic interference shielding studies of nickel oxide and tungsten oxide reinforced polyvinylchloride nanocomposites. Polym Technol Mater.

[41] Rani P, Ahamed MB, Deshmukh K (2021). Structural, dielectric and EMI shielding properties of polyvinyl alcohol/chitosan blend nanocomposites integrated with graphite oxide and nickel oxide nanofillers. J Mater Sci Mater Electron.

[42] Dhatarwal P, Sengwa RJ (2020). Structural and dielectric characterization of (PVP/PEO)/Al_2_O_3_ nanocomposites for biodegradable nanodielectric applications. Adv Compos Hybrid Mater.

[43] Saied MA, Ward AA (2020). Physical, dielectric and biodegradation studies of PVC/silica nanocomposites based on traditional and environmentally friendly plasticizers. Adv Nat Sci Nanosci Nanotechnol.

[44] Dhatarwal P, Choudhary S, Sengwa RJ (2020). Effectively nanofiller concentration tunable dielectric properties of PVP/SnO_2_ nanodielectrics. Mater Lett.

[45] Chatterjee B, Kulshrestha N, Gupta PN (2016). Nano composite solid polymer electrolytes based on biodegradable polymers starch and poly vinyl alcohol. Meas J Int Meas Confed.

[46] Fal J, Bulanda K, Oleksy M, Sobczak J, Shi J, Liu M, Graphite – Diamond Polylactide Nanocomposites, (2021) 10.3390/ma14112835 PMC8198477 34073172

[47] Xu W, Raychowdhury S, Jiang DD, Retsos H, Giannelis EP (2008). Dramatic improvements in toughness in poly(lactide-co-glycolide) nanocomposites. Small.

[48] Dey KK, Kumar P, Yadav RR, Dhar A, Srivastava AK (2014). CuO nanoellipsoids for superior physicochemical response of biodegradable PVA. RSC Adv.

[49] Rostami F, Tamjid E, Behmanesh M (2020). Drug-eluting PCL/graphene oxide nanocomposite scaffolds for enhanced osteogenic differentiation of mesenchymal stem cells. Mater Sci Eng C.

[50] Hammami I, Benhamou K, Hammami H, SoretoTeixeira S, Arous M, Kaddami H, Graça MPF, Costa LC (2020). Electrical, morphology and structural properties of biodegradable nanocomposite polyvinyl-acetate/ cellulose nanocrystals. Mater Chem Phys.

[51] Wu W, Liu T, Zhang D, Sun Q, Cao K, Zha J, Lu Y, Wang B, Cao X, Feng Y, Roy VAL, Li RKY (2019). Significantly improved dielectric properties of polylactide nanocomposites via TiO_2_ decorated carbon nanotubes. Compos Part A Appl Sci Manuf.

[52] Lao J, Xie H, Shi Z, Li G, Li B, Hu GH, Yang Q, Xiong C (2018). Flexible regenerated cellulose/boron nitride nanosheet high-temperature dielectric nanocomposite films with high energy density and breakdown strength. ACS Sustain Chem Eng.

[53] Kashi S, Gupta RK, Baum T, Kao N, Bhattacharya SN (2016). Dielectric properties and electromagnetic interference shielding effectiveness of graphene-based biodegradable nanocomposites. Mater Des.

[54] Zhang H, Peng M, Cheng T, Zhao P, Qiu L, Zhou J, Lu G, Chen J (2018). Silver nanoparticles-doped collagen–alginate antimicrobial biocomposite as potential wound dressing. J Mater Sci.

[55] Jeong W, Shin S (2022). Processing in materials processing biocompatibility and corrosion behavior of heat-treated Ti6Al4V-equine bone nanocomposites. J Mater Sci.

[56] Inal S, Rivnay J, Suiu AO, Malliaras GG, McCulloch I (2018). Conjugated polymers in bioelectronics. Acc Chem Res.

[57] Salehi MH, Golbaten-Mofrad H, Jafari SH, Goodarzi V, Entezari M, Hashemi M, Zamanlui S (2021). Electrically conductive biocompatible composite aerogel based on nanofibrillated template of bacterial cellulose/polyaniline/nano-clay. Int J Biol Macromol.

[58] Golbaten-mofrad H, Seyfi A, Seyfikar S, Hadi M, Goodarzi V, Wurm FR, Hassan S (2021). Facile template preparation of novel electroactive scaffold composed of polypyrrole-coated poly ( glycerol-sebacate-urethane ) for tissue engineering applications. Eur Polym J.

[59] Ghaffari-bohlouli P, Golbaten-mofrad H, Najmoddin N (2023). Reinforced conductive polyester based on itaconic acids, glycerol and polypyrrole with potential for electroconductive tissue restoration. Synth Met.

[60] Torabi A, Hassan S, Hossein J, Khonakdar A, Goodarzi V, Yu L (2022). Development of electroactive nanocomposites based on poly ( vinylidene fluoride - hexafluoropropylene )/ polycarbonate blends with improved dielectric, thermal, and mechanical properties. J Polym Res.

[61] Panahi-sarmad M, Noroozi M, Abrisham M, Eghbalinia S, Teimoury F, Bahramian AR, Dehghan P, Sadri M, Goodarzi V (2020). A comprehensive review on carbon-based polymer nanocomposite foams as electromagnetic interference shields and piezoresistive sensors. ACS Appl Electr Mater.

[62] Zarrintaj P, Urbanska AM, Seyed S, Goodarzi V, Reza M, Mozafari M (2018). A facile route to the synthesis of anilinic electroactive colloidal hydrogels for neural tissue engineering applications. J Colloid Interface Sci.

[63] Pandey M, Joshi GM, Deshmukh K, Ahmad J (2015). Impedance spectroscopy and conductivity studies of CdCl_2_ doped polymer electrolyte. Adv Mater Lett.

[64] Abutalib MM, Rajeh A (2020). Structural, thermal, optical and conductivity studies of Co/ZnO nanoparticles doped CMC polymer for solid state battery applications. Polym Test.

[65] Cho J, Lee H, Nam KH, Yeo H, Yang CM, Seong DG, Lee D, Kim SY (2020). Enhanced electrical conductivity of polymer nanocomposite based on edge-selectively functionalized graphene nanoplatelets. Compos Sci Technol.

[66] Alves Z, Ferreira NM, Mendo S, Ferreira P, Nunes C (2021). Design of alginate-based bionanocomposites with electrical conductivity for active food packaging. Int J Mol Sci.

[67] Bin-Dahman OA, Rahaman M, Khastgir D, Al-Harthi MA (2018). Electrical and dielectric properties of poly(vinyl alcohol)/starch/graphene nanocomposites. Can J Chem Eng.

[68] Kim H, Kobayashi S, Abdurrahim MA, Zhang MJ, Khusainova A, Hillmyer MA, Abdala AA, MacOsko CW (2011). Graphene/polyethylene nanocomposites: effect of polyethylene functionalization and blending methods. Polymer (Guildf).

[69] Aguilar JO, Bautista-Quijano JR, Avilés F (2010). Influence of carbon nanotube clustering on the electrical conductivity of polymer composite films. Express Polym Lett.

[70] Nezakati T, Tan A, Lim J, Cormia RD, Teoh SH, Seifalian AM (2019). Ultra-low percolation threshold POSS-PCL/graphene electrically conductive polymer: Neural tissue engineering nanocomposites for neurosurgery. Mater Sci Eng C.

[71] Spinelli G, Lamberti P, Tucci V, Kotsilkova R, Ivanov E, Menseidov D, Naddeo C, Romano V, Guadagno L, Adami R, Meisak D, Bychanok D, Kuzhir P (2019). Nanocarbon/poly(lactic) acid for 3D printing: effect of fillers content on electromagnetic and thermal properties. Materials (Basel).

[72] Jahan N (2020). Temperature dependence of electrical properties of biopolymer gel. Polymer Concentrat.

[73] Grossiord N, Kivit PJJ, Loos J, Meuldijk J, Kyrylyuk AV, van der Schoot P, Koning CE (2008). On the influence of the processing conditions on the performance of electrically conductive carbon nanotube/polymer nanocomposites. Polymer (Guildf).

[74] Kochetov R, Korobko AV, Andritsch T, Morshuis PHF, Picken SJ, Smit JJ (2011). Modelling of the thermal conductivity in polymer nanocomposites and the impact of the interface between filler and matrix. J Phys D Appl Phys.

[75] Han Z, Fina A (2011). Thermal conductivity of carbon nanotubes and their polymer nanocomposites: a review. Prog Polym Sci.

[76] Eichhorn SJ, Etale A, Wang J, Berglund LA, Li Y, Cai Y, Chen C, Cranston ED, Johns MA, Fang Z, Li G, Hu L, Khandelwal M, Lee KY, Oksman K, Pinitsoontorn S, Quero F, Sebastian A, Titirici MM, Xu Z, Vignolini S, Frka-Petesic B (2022). Current international research into cellulose as a functional nanomaterial for advanced applications. J Mater Sci.

[77] Silva F, Gracia N, McDonagh BH, Domingues FC, Nerín C, Chinga-Carrasco G (2019). Antimicrobial activity of biocomposite films containing cellulose nanofibrils and ethyl lauroyl arginate. J Mater Sci.

[78] J. Sun, Q. Xue, Electrical conductivity and percolation behavior of polymer nanocomposites, 2016. https://www.springer.com/gb/book/9783319282367#aboutBook.

[79] Zhao M, Zhou Y, Li X, Cheng W, Zhou C, Ma T (2020). remote sensing of environment mapping urban dynamics (1992–2018) in Southeast Asia using consistent nighttime light data from DMSP and VIIRS. Remote Sens Environ.

[80] Kong L, Liu G (2021). Synchrotron-based infrared microspectroscopy under high pressure: an introduction. Matter Radiat Extr.

[81] Huang C, Qian X, Yang R (2018). Thermal conductivity of polymers and polymer nanocomposites. Mater Sci Eng R Reports.

[82] Yang S, Huang Z, Hu Q, Zhang Y, Wang F, Wang H (2022). Proportional optimization model of multiscale spherical BN for enhancing thermal conductivity. ACS Appl Electr Mater.

[83] Kuang W, Wang H, Li X, Zhang J, Zhou Q, Zhao Y (2018). Acta Materialia Application of the thermodynamic extremal principle to diffusion- controlled phase transformations in Fe-C-X alloys: modeling and applications. Acta Mater.

[84] Alexandre M, Dubois P (2000). Polymer-layered silicate nanocomposites: preparation, properties and uses of a new class of materials. Mater Sci Eng R Reports.

[85] Kumar AP, Depan D, Singh Tomer N, Singh RP (2009). Nanoscale particles for polymer degradation and stabilization-Trends and future perspectives. Prog Polym Sci.

[86] Glaskova-Kuzmina T, Starkova O, Gaidukovs S, Platnieks O, Gaidukova G (2021). Durability of biodegradable polymer nanocomposites. Polymers (Basel).

[87] Idumah CI, Hassan A (2016). Recently emerging trends in thermal conductivity of polymer nanocomposites. Rev Chem Eng.

[88] Guo Y, Zuo X, Xue Y, Tang J, Gouzman M, Fang Y, Zhou Y, Wang L, Yu Y, Rafailovich MH (2020). Engineering thermally and electrically conductive biodegradable polymer nanocomposites. Compos Part B Eng.

[89] Nilagiri Balasubramanian KB, Ramesh T (2018). Role, effect, and influences of micro and nano-fillers on various properties of polymer matrix composites for microelectronics: a review. Polym Adv Technol.

[90] Franco-Bacca AP, Cervantes-Alvarez F, Macías JD, Castro-Betancur JA, Pérez-Blanco RJ, Giraldo Osorio OH, Arias Duque NP, Rodríguez-Gattorno G, Alvarado-Gil JJ (2021). Heat transfer in cassava starch biopolymers: effect of the addition of Borax. Polymers.

[91] Gumede TP, Luyt AS, Tercjak A, Müller AJ (2019). Isothermal crystallization kinetics and morphology of double crystalline PCL/PBS blends mixed with a polycarbonate/MWCNTs masterbatch. Polymers (Basel).

[92] Burger N, Laachachi A, Ferriol M, Lutz M, Toniazzo V, Ruch D (2016). Review of thermal conductivity in composites: Mechanisms, parameters and theory. Prog Polym Sci.

[93] Chen H, Ginzburg VV, Yang J, Yang Y, Liu W, Huang Y, Du L, Chen B (2016). Thermal conductivity of polymer-based composites: Fundamentals and applications. Prog Polym Sci.

[94] Leung SN (2018). Thermally conductive polymer composites and nanocomposites: processing-structure-property relationships. Compos Part B Eng.

[95] Armentano I, Dottori M, Fortunati E, Mattioli S, Kenny JM (2010). Biodegradable polymer matrix nanocomposites for tissue engineering: a review. Polym Degrad Stab.

[96] Huang X, Zhi C (2016). Polymer nanocomposites: electrical and thermal properties. Polym Nanocomp Electr Thermal Prop.

[97] Pielichowski K, Leszczynska A, Njuguna J (2010). Mechanisms of thermal stability enhancement in polymer nanocomposites. Optim Polym Nanocomp Prop.

[98] Moniruzzaman M, Winey KI (2006). Polymer nanocomposites containing carbon nanotubes. Macromolecules.

[99] Yang J, Lin Y, Wang J, Lai M, Li J, Liu J, Tong X, Cheng H (2005). Morphology, thermal stability, and dynamic mechanical properties of atactic polypropylene/carbon nanotube composites. J Appl Polym Sci.

[100] Marosföi BB, Marosi GJ, Szép A, Anna P, Keszei S, Nagy BJ, Martvonova H, Sajó IE (2006). Complex activity of clay and CNT particles in flame retarded EVA copolymer. Polym Adv Technol.

[101] Chipara M, Lozano K, Hernandez A, Chipara M (2008). TGA analysis of polypropylene e carbon nanofibers composites. Polymer Degrad Stabil.

[102] Di Blasi C, Galgano A, Branca C (2013). Modeling the thermal degradation of poly(methyl methacrylate)/carbon nanotube nanocomposites. Polym Degrad Stab.

[103] Chen X, Wang J, Lin M, Zhong W, Feng T, Chen X, Chen J, Xue F (2008). Mechanical and thermal properties of epoxy nanocomposites reinforced with amino-functionalized multi-walled carbon nanotubes. Mater Sci Eng A.

[104] Bikiaris D (2011). Can nanoparticles really enhance thermal stability of polymers? Part II: an overview on thermal decomposition of polycondensation polymers. Thermochim Acta.

[105] Kornai A (2008). The elements. Adv Inf Knowl Process.

[106] Pandey P, Mohanty S, Nayak SK (2014). Improved flame retardancy and thermal stability of polymer/clay nanocomposites, with the incorporation of multiwalled carbon nanotube as secondary filler: evaluation of hybrid effect of nanofillers. High Perform Polym.

[107] Sownthari K, Suthanthiraraj SA (2015). Preparation and properties of biodegradable polymer-layered silicate nanocomposite electrolytes for zinc based batteries. Electrochim Acta.

[108] Remiš T (2021). properties of nanodiamond-reinforced poly ( vinyl alcohol) nanocomposites. Polymers.

[109] Sabater i Serra R, Molina-Mateo J, Torregrosa-Cabanilles C, Andrio-Balado A, Dueñas JMM, Serrano-Aroca Á (2020). Bio-Nanocomposite hydrogel based on zinc alginate/graphene oxide: morphology, structural conformation, thermal behavior/degradation, and dielectric properties. Polymers (Basel).

[110] Silva MM, Lopes PE, Li Y, Pötschke P, Ferreira FN, Paiva MC (2021). Polylactic acid/carbon nanoparticle composite filaments for sensing. Appl Sci.

[111] Lule Z, Kim J (2019). Surface modification of aluminum nitride to fabricate thermally conductive poly (Butylene Succinate) nanocomposite. Polymers (Basel).

[112] Chen BK, Shih CC, Chen AF (2012). Ductile PLA nanocomposites with improved thermal stability. Compos Part A Appl Sci Manuf.

[113] Mofokeng JP, Luyt AS (2015). Morphology and thermal degradation studies of melt-mixed PLA/PHBV biodegradable polymer blend nanocomposites with TiO_2_ as filler. J Appl Polym Sci.

[114] Botana A, Mollo M, Eisenberg P, Torres Sanchez RM (2010). Effect of modified montmorillonite on biodegradable PHB nanocomposites. Appl Clay Sci.

[115] Behera K, Chang YH, Yadav M, Chiu FC (2020). Enhanced thermal stability, toughness, and electrical conductivity of carbon nanotube-reinforced biodegradable poly(lactic acid)/poly(ethylene oxide) blend-based nanocomposites. Polymer (Guildf).

[116] Sivanjineyulu V, Behera K, Chang Y-H, Chiu F-C (2018). Selective localization of carbon nanotube and organoclay in biodegradable poly(butylene succinate)/polylactide blend-based nanocomposites with enhanced rigidity, toughness and electrical conductivity. Compos. Part A Appl. Sci. Manuf..

[117] Khalili MA, Tamjid E (2021). Controlled biodegradation of magnesium alloy in physiological environment by metal organic framework nanocomposite coatings. Sci Rep.

[118] Fukushima K, Tabuani D, Abbate C, Arena M, Rizzarelli P (2011). Preparation, characterization and biodegradation of biopolymer nanocomposites based on fumed silica. Eur Polym J.

[119] Okamoto M, John B (2013). Synthetic biopolymer nanocomposites for tissue engineering scaffolds. Prog Polym Sci.

[120] Dias OAT, Konar S, Leão AL, Yang W, Tjong J, Sain M (2020). Current state of applications of nanocellulose in flexible energy and electronic devices. Front Chem.

[121] Kolonelou E, Loupou E, Klonos PA, Sakellis E, Valadorou D, Kyritsis A, Papathanassiou AN (2021). Thermal and electrical characterization of poly(vinyl)alcohol)/poly(vinylidene fluoride) blends reinforced with nano-graphene platelets. Polymer (Guildf).

[122] Li A, Zhang C, Zhang YF (2017). Thermal conductivity of graphene-polymer composites: mechanisms, properties, and applications. Polymers (Basel).

[123] Behera K, Chang Y-H, Yadav M, Chiu F-C (2020). Enhanced thermal stability, toughness, and electrical conductivity of carbon nanotube-reinforced biodegradable poly(lactic acid)/poly(ethylene oxide) blend-based nanocomposites. Polymer (Guildf)..

[124] Kamal A, Ashmawy M, Shanmugan S, Algazzar AM, Elsheikh AH (2022). Fabrication techniques of polymeric nanocomposites: a comprehensive review. Proceed Instit Mech Eng, Part C: J Mech Eng Sci.

[125] Pielichowski K, Pielichowska K (2018). Polymer nanocomposites.

[126] Tanahashi M (2010). Development of fabrication methods of filler/polymer nanocomposites: with focus on simple melt-compounding-based approach without surface modification of nanofillers. Materials.

[127] Panahi Z, Tamjid E, Rezaei M (2020). Surface modification of biodegradable AZ91 magnesium alloy by electrospun polymer nanocomposite: Evaluation of in vitro degradation and cytocompatibility. Surf Coatings Technol.

[128] Mittal V, Characterization of nanocomposite materials : an overview, (2012) 1–12.

[129] D. Son, S. Cho, J. Nam, H. Lee, M. Kim, X-ray-Based spectroscopic techniques for characterization of polymer nanocomposite materials at a molecular level, (n.d.). 10.3390/polym12051053 PMC7284789 32375363

[130] Cao Y, Uhrich KE (2019). Biodegradable and biocompatible polymers for electronic applications: a review. J Bioact Compat Polym.

[131] Tamjid E, Bohlouli M, Mohammadi S, Alipour H, Nikkhah M (2020). Sustainable drug release from highly porous and architecturally engineered composite scaffolds prepared by 3D printing. J Biomed Mater Res - Part A.

[132] Nojoomi A, Tamjid E, Simchi A, Bonakdar S, Stroeve P (2017). Injectable polyethylene glycol-laponite composite hydrogels as articular cartilage scaffolds with superior mechanical and rheological properties. Int J Polym Mater Polym Biomater.

[133] Shafei S, Foroughi J, Stevens L, Wong CS, Zabihi O, Naebe M (2017). Electroactive nanostructured scaffold produced by controlled deposition of PPy on electrospun PCL fibres. Res Chem Intermed.

[134] Chen C, Tang Y, Ye YS, Xue Z, Xue Y, Xie X, Mai YW (2014). High-performance epoxy/silica coated silver nanowire composites as underfill material for electronic packaging. Compos Sci Technol.

[135] Boutry CM, Kaizawa Y, Schroeder BC, Chortos A, Legrand A, Wang Z, Chang J, Fox P, Bao Z (2018). A stretchable and biodegradable strain and pressure sensor for orthopaedic application. Nat Electron.

[136] Dagdeviren C, Hwang SW, Su Y, Kim S, Cheng H, Gur O, Haney R, Omenetto FG, Huang Y, Rogers JA (2013). Transient, biocompatible electronics and energy harvesters based on ZnO. Small.

[137] Li R, Wang L, Yin L (2018). Materials and devices for biodegradable and soft biomedical electronics. Materials (Basel).

[138] Feig VR, Tran H, Bao Z (2018). Biodegradable polymeric materials in degradable electronic devices. ACS Cent Sci.

[139] Kausar A (2020). A review of high performance polymer nanocomposites for packaging applications in electronics and food industries. J Plast Film Sheeting.

[140] Sun J, Li H, Huang Y, Zheng X, Liu Y, Zhuang J, Wu D (2019). Simple and affordable way to achieve polymeric superhydrophobic surfaces with biomimetic hierarchical roughness. ACS Omega.

[141] Pandey M, Joshi GM, Bhattacharya S (2016). Influence of K_2_Ti_6_O_13_ on dielectric and barrier properties of polymer. Ionics (Kiel).

[142] Khutia M, Joshi GM, Deshmukh K, Pandey M (2015). Preparation of modified polymer blend and electrical performance, Compos. Interfaces.

[143] Lokanatha Reddy P, Deshmukh K, Chidambaram K, Ahamed B, Sadasivuni KK, Ponnamma D, Lakshmipathy R, Dayananda D, Khadheer Pasha SK (2019). Effect of poly ethylene glycol (PEG) on structural, thermal and photoluminescence properties of CdO nanoparticles for optoelectronic applications. Mater Today Proc.

[144] Mangaraj S, Yadav A, Bal LM, Dash SK, Mahanti NK (2019). Application of biodegradable polymers in food packaging industry: a comprehensive review. J Packag Technol Res.

